# Multimodal fusion-powered English speaking robot

**DOI:** 10.3389/fnbot.2024.1478181

**Published:** 2024-11-15

**Authors:** Ruiying Pan

**Affiliations:** The College of Henan Procuratorial Profession, Zhengzhou, China

**Keywords:** ALBEF, Neural Machine Translation (NMT), cross-attention mechanism, multimodal robot, speech recognition

## Abstract

**Introduction:**

Speech recognition and multimodal learning are two critical areas in machine learning. Current multimodal speech recognition systems often encounter challenges such as high computational demands and model complexity.

**Methods:**

To overcome these issues, we propose a novel framework-EnglishAL-Net, a Multimodal Fusion-powered English Speaking Robot. This framework leverages the ALBEF model, optimizing it for real-time speech and multimodal interaction, and incorporates a newly designed text and image editor to fuse visual and textual information. The robot processes dynamic spoken input through the integration of Neural Machine Translation (NMT), enhancing its ability to understand and respond to spoken language.

**Results and discussion:**

In the experimental section, we constructed a dataset containing various scenarios and oral instructions for testing. The results show that compared to traditional unimodal processing methods, our model significantly improves both language understanding accuracy and response time. This research not only enhances the performance of multimodal interaction in robots but also opens up new possibilities for applications of robotic technology in education, rescue, customer service, and other fields, holding significant theoretical and practical value.

## 1 Introduction

The research on English speech recognition technology holds significant practical importance and broad application prospects (Kheddar et al., 2024). It not only enhances the naturalness and efficiency of human-computer interaction but also plays a crucial role in fields such as education, healthcare, and smart homes. Furthermore, the advancement of speech recognition technology can provide convenient communication means for individuals with hearing impairments, thereby improving their quality of life (Al-Fraihat et al., 2024). In the context of accelerating globalization, cross-language communication has become increasingly frequent. As English is a global lingua franca, the refinement and application of its speech recognition technology can not only facilitate international communication but also promote the integration and advancement of science and culture among nations. Therefore, the research and development of English speech recognition technology are not only a critical aspect of technological innovation but also have profound implications for social progress and human well-being (Dhanjal and Singh, 2024).

Traditional methods for English speech recognition primarily involve symbolic AI and knowledge representation. These methods include expert systems, rule-based approaches, and frame-based approaches. Expert systems use the knowledge of domain experts to perform reasoning and decision-making. They achieve speech recognition functionality through the construction of knowledge bases and inference engines. For example, the DENDRAL project used expert systems for chemical structure analysis and successfully applied similar techniques in speech recognition (Yang and Zhu, 2024). Another example is the MYCIN system, which supports clinical decision-making through expert systems and has shown similar potential in speech recognition (Tarasiev et al., 2024). Rule-based approaches map speech signals to corresponding text using a set of explicit rules. For instance, speech signals are converted into sentence structures through a series of grammatical rules (Chen, 2024). The ELIZA program, which uses a predefined set of rules for simple natural language processing, is another example where rule-based methods have been applied in speech recognition (Yan et al., 2024). Frame-based approaches use structured frameworks to represent knowledge and perform reasoning and recognition through the relationships between these frameworks. For example, frame nets were used to construct the knowledge representation structure of speech recognition systems, interpreting speech signals through the relationships between frames (Yeo et al., 2024). The STRIPS system, which uses frame nets for problem-solving, also demonstrated potential applications in speech recognition (Wang et al., 2024b). These methods offer advantages such as clear knowledge representation and transparent reasoning processes. However, they have limitations, including the inability to handle complex and variable speech environments effectively and the time-consuming and labor-intensive nature of constructing knowledge bases.

To address the limitations of traditional algorithms in handling complex and variable speech environments, data-driven and machine learning algorithms have been employed in English speech recognition. These methods solve the problem by utilizing large datasets to train and optimize models, offering advantages such as strong adaptability and high accuracy. For instance, decision tree-based methods use binary tree structures for classification and regression to achieve efficient speech recognition. A typical application is differentiating between various speech signals through decision tree construction (Wang, 2024). Another example is the use of Classification and Regression Trees (CART) algorithm to handle complex data in speech recognition (Raju and Kumari, 2024). Random forest methods improve classification accuracy and robustness by constructing multiple decision trees and aggregating their votes. This approach demonstrates superior speech recognition performance. For example, random forest algorithms combine multiple classification results to enhance overall recognition rates (Rokach, 2016). Another example is the use of random forests to show high robustness in processing speech signals in noisy environments (Reddy and Pachori, 2024). Support Vector Machines (SVM) classify data by constructing hyperplanes in high-dimensional spaces, effectively handling nonlinear speech data. For example, SVM is used to distinguish different phonemes in speech signals (Cedeno-Moreno et al., 2024). Another example is improving recognition accuracy and efficiency by processing large volumes of speech data with SVM (Kanisha et al., 2024). However, these methods have the drawbacks of long model training times and high computational resource requirements.

To address the issues of long model training times and high computational resource demands in statistical and machine learning-based speech recognition, deep learning algorithms have been employed in English speech recognition. These methods primarily involve constructing multi-layer neural networks and integrating multimodal data, offering advantages such as automatic feature extraction and strong capability to handle complex data. For instance, Convolutional Neural Networks (CNNs) excel in feature extraction and classification of speech signals through hierarchical structures. They have shown outstanding performance in speech recognition tasks, such as classifying spectrograms of speech signals using CNNs (Ilgaz et al., 2024). Another example is the use of deep CNNs to process large amounts of speech data, which improves accuracy and efficiency in speech recognition (Abdel-Hamid et al., 2014). Reinforcement learning methods optimize model strategies through reward mechanisms, demonstrating good adaptability in dynamic and complex environments. For example, reinforcement learning can be used to optimize parameter configurations in speech recognition systems (Yang et al., 2024). Another example is applying deep reinforcement learning to continuous speech recognition tasks (Li et al., 2016). Transformer models handle sequence data through self-attention mechanisms and have shown excellent performance in speech recognition. For instance, Transformer models achieve efficient sequence-to-sequence speech conversion (Ryumin et al., 2024). Another example is using the Transformer architecture to improve the robustness and accuracy of speech recognition (Bahdanau et al., 2016). However, these methods come with the drawbacks of high computational resource consumption and increased model complexity.

To address the issues of high computational resource consumption and model complexity, we propose our approach: EnglishAL-Net: a Multimodal English Speaking Robot Driven by Neural Machine Translation. Traditional speech recognition methods face several limitations, such as limited ability to handle complex and variable speech environments, time-consuming and labor-intensive knowledge base construction, and high computational resource demands. The ALBEF (Align Before Fuse) model significantly reduces model complexity and computational requirements by aligning multimodal information before fusion, addressing the excessive computational overhead caused by modality fusion in traditional methods. The NMT (Neural Machine Translation) model has significant advantages in language conversion and generation, effectively tackling the non-linearity and complexity challenges in speech signals. Combining these two approaches enables more effective English oral communication for robots. Additionally, the cross-attention mechanism further enhances the accuracy and robustness of speech recognition and generation by establishing associations between different modalities, solving the problem of low recognition accuracy due to insufficient information sharing between modalities in traditional methods. The motivation behind this research is that current speech recognition systems perform poorly in complex environments and have high computational demands. By combining multimodal data and advanced attention mechanisms, we can significantly improve system performance and application scope, thus providing stronger support for natural communication between robots and humans.

- The EnglishAL-Net is introduced combined with the cross-attention mechanism to align multi-modal information before fusion, significantly reducing model complexity and computing requirements.
- This method performs well in multiple scenarios, is efficient and versatile, and can achieve high-precision speech recognition in both noisy and quiet environments.
- Experimental results show that the system using this method is significantly better than the traditional method in terms of speech recognition accuracy and robustness, and still maintains efficient performance in complex environments.

## 2 Related work

### 2.1 Speech recognition

The origins of speech recognition technology date back to the 1950s, but significant progress has been made in recent decades, largely due to advancements in deep learning and big data. Early systems relied on finite state machines and Hidden Markov Models (HMMs) (Rabiner, 1989), which were effective for small-scale (Voß et al., 2024), domain-specific tasks but struggled with complex and large-scale speech data. With the advent of the 21st century, machine learning technologies like Support Vector Machines (SVMs) and Artificial Neural Networks (ANNs) began to transform the field. The introduction of Deep Neural Networks (DNNs), including Convolutional Neural Networks (CNNs) and Recurrent Neural Networks (RNNs), greatly enhanced speech recognition performance. RNNs, along with Long Short-Term Memory (LSTM) networks and Gated Recurrent Units (GRUs), improved accuracy by capturing time-series data dependencies (Zhu et al., 2024). Recently, Transformer models, known for their attention mechanisms, have brought new advancements to speech recognition. Models like DeepSpeech and wav2vec 2.0, based on Transformers, excel even in noisy or resource-constrained environments. The rise of end-to-end speech recognition methods has further simplified the process by removing the need for complex feature engineering. These methods use a unified neural network architecture to convert raw audio into text, streamlining system design and optimization (Jin et al., 2024b).

### 2.2 Multimodality

The development of multimodal technology aims to replicate how humans naturally understand and process information by integrating data from different sensory modalities (such as vision, hearing, touch, etc.), enhancing machine perception and decision-making capabilities. Early multimodal research primarily focused on optimizing individual modality performance, often overlooking the synergistic effects of integrating multiple modalities (Jin et al., 2024a). With advancements in computational power and the widespread use of deep learning, multimodal technology has begun to show significant potential. Convolutional neural networks (CNN) and recurrent neural networks (RNN) have made notable progress in handling visual and language information, enabling cross-modal information fusion. For example, tasks like image captioning and visual question answering combine visual and language data to automatically generate image descriptions and answer questions based on image content (Wang et al., 2021a). Recently, the introduction of transformer models has further advanced multimodal technology. Transformer models, based on attention mechanisms, can simultaneously process and integrate information from different modalities, such as visual-language pre-training models (VLP) and BERT4Video for video understanding. These models, through pre-training and fine-tuning, excel in multiple multimodal tasks, demonstrating strong cross-modal understanding and generation capabilities (Jingning, 2024). Moreover, multimodal technology has made significant strides in practical applications. In autonomous driving, the fusion of multimodal sensor data (such as lidar, cameras, and radar) enhances environmental perception accuracy and robustness. In medical diagnostics, multimodal analysis combining imaging, pathology, and genetic data provides more comprehensive and precise diagnostic results (Wang et al., 2021b). Looking ahead, the development of multimodal technology will focus on more efficient cross-modal fusion methods, larger-scale pre-training models, and more versatile and flexible multimodal systems. These advancements will further drive the application of multimodal technology in natural language processing, computer vision, robotics, and other fields, enabling machines to understand and interact with complex multimodal information more naturally and intelligently (Chen et al., 2024).

### 2.3 Cross-attention mechanisms

Cross-attention mechanisms are crucial in deep learning for enhancing model performance and generalization by creating dynamic associations between different modalities or levels. Their applications span various fields, including natural language processing, computer vision, and multimodal fusion. In natural language processing, cross-attention is vital for tasks like machine translation and text generation. In transformer models, cross-attention layers enable flexible focus on different parts of the encoder's output during decoding, leading to smoother and more accurate translations. Pre-trained language models such as BERT and GPT also use cross-attention to capture and utilize contextual information (Prasangini and Nagahamulla, 2018), significantly boosting performance across various NLP tasks (Koyama et al., 2020). In computer vision, cross-attention mechanisms are used in object detection, image segmentation, and image generation. For example, they help models identify and locate objects in complex scenes more accurately, and Vision Transformers (ViT) use attention between image patches to improve feature extraction compared to traditional convolutional neural networks. Multimodal fusion benefits from cross-attention by integrating and complementing information across different modalities, such as images, text, and audio (Jin et al., 2023). In visual question answering (VQA) and image captioning, cross-attention mechanisms allow models to focus on relevant parts of both questions and images or regions of images while generating text. Additionally, cross-attention shows promise in recommendation systems and medical diagnostics. It can link user and item features for more personalized recommendations and integrate imaging, genetic, and clinical data to enhance diagnostic accuracy and reliability.

## 3 Method

### 3.1 Overview of our network

**Figure 1:**
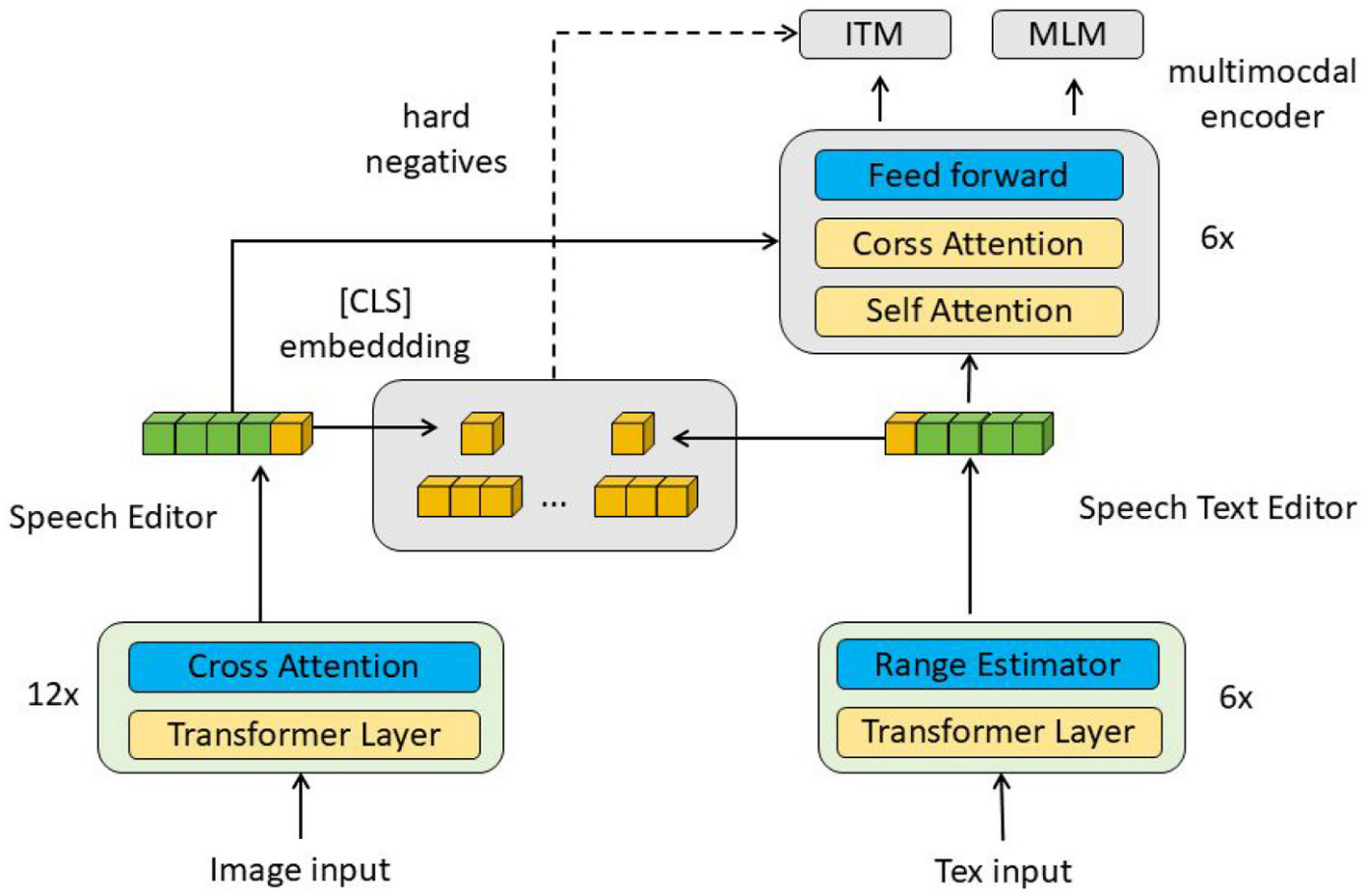
Overall framework diagram. The data flow in the figure starts from the image and text input, and is processed by multiple layers of cross-attention and transformer respectively, and then fused through the multimodal encoder, and the final output is used for ITM and MLM tasks.

In this paper, the proposed EnglishAL-Net model enhances the robot's ability to process visual and auditory inputs by leveraging Neural Machine Translation (NMT) (as shown in Figure 1). EnglishAL-Net combines the strengths of the ALBEF-NMT model and a cross-attention mechanism to improve semantic understanding. ALBEF (Align before Fuse) is a multimodal learning method that aligns and fuses data, particularly suited for visual and textual inputs. This approach processes each modality's data separately before using alignment mechanisms to ensure high semantic consistency between different modalities. Finally, the fused data is further processed, enabling EnglishAL-Net to understand and respond more accurately to complex spoken instructions in various environments.

EnglishAL-Net improves the speed of autoregressive correction models by using a non-autoregressive (NAR) generation technique coupled with alignment-based editing. Initially, it calculates the Levenshtein distance between the source sentence (recognized text) and the target sentence (reference text). By analyzing the insertions, deletions, and substitutions indicated by the Levenshtein distance, it determines the number of target tokens corresponding to each source token post-editing. Specifically, a deletion is marked by 0, no change or substitution by 1, and an insertion by values of 2 or more. EnglishAL-Net employs a non-autoregressive (NAR) encoder-decoder structure and incorporates a range estimator to manage length discrepancies between the source sequence (encoder) and the target sequence (decoder). This range estimator is trained to adjust each source token as necessary, ensuring the correct number of target tokens. These adjusted source tokens are then processed by the decoder in parallel. Subsequent sections will discuss the alignment-based editing, model architecture, and pre-training methods used in EnglishAL-Net.

The ALBEF (Align Before Fuse) model was optimized by focusing on the alignment of multimodal data before fusion, reducing computational overhead and improving real-time performance. Specifically, we fine-tuned the cross-attention layers to ensure that the alignment between text and visual inputs is efficient. We implemented dynamic weight adjustments to prioritize high-confidence modality inputs, which helps the model process ambiguous or noisy data more effectively. This optimization significantly reduces computational costs, especially in scenarios with large, complex datasets.

The newly designed text and image editor integrates seamlessly with the ALBEF model by leveraging alignment-based mechanisms. This editor is built to support dynamic editing of both textual and visual content in real-time, ensuring that contextual and semantic relationships between modalities are preserved. It works by first segmenting both text and images into meaningful units (words, phrases, image regions) and then applying cross-modal attention to match corresponding elements. We also incorporated an error detection and correction mechanism based on Levenshtein distance, allowing the editor to automatically refine outputs based on alignment quality between the recognized input and reference data.

In EnglishAL-Net, multimodal data–such as text and image inputs–are integrated through an optimized version of the ALBEF (Align Before Fuse) model. The key steps in this integration process are as follows: Initially, the input modalities (text and image) are processed separately through dedicated encoders. For text, we use a transformer-based encoder that captures the semantic information from the input. For images, a convolutional neural network (CNN) is used to extract visual features, generating a feature map that represents different parts of the image. After encoding, the ALBEF model ensures that the features from both modalities are aligned before they are fused. This alignment is crucial for preserving the semantic relationship between the text and image data. Specifically, we use a cross-attention mechanism that aligns the relevant parts of the image to the corresponding text elements, ensuring that the information from both modalities is contextually related before fusion. The cross-attention layers allow the model to focus on the relevant parts of each modality that are most informative for the task at hand. For example, when processing a spoken command alongside an image, the cross-attention mechanism identifies which part of the image is relevant to the spoken words and aligns them. This dynamic attention helps reduce irrelevant information and emphasizes the meaningful connections between modalities. Once the alignment is completed, the fused multimodal representation is formed by combining the aligned text and image features. The fused representation is then processed through the remaining network layers, allowing EnglishAL-Net to generate a response or perform the required task with a deeper understanding of the multimodal input. After the fusion, the multimodal representation is fed into a neural machine translation (NMT) module to generate the final output. This step is particularly useful for tasks like speech recognition and response generation, where the output is based on the combined understanding of both text and visual inputs. Additionally, a real-time text and image editor refines the results, ensuring higher precision and coherence. This structured approach to multimodal integration significantly improves the model's ability to handle complex tasks that involve both language and visual data, ensuring that EnglishAL-Net can robustly process and respond to a wide range of inputs. The combination of alignment before fusion and cross-attention enables the model to make more accurate associations between modalities, resulting in enhanced performance across different domains.

### 3.2 Edit alignment

#### 3.2.1 Calculating edit path

Edit distance is a metric used to measure the difference between two sentences by calculating the minimum number of edit operations required to transform the source sentence into the target sentence. These operations include inserting, deleting, and substituting tokens. Assume that the source sentence is *X* = (*x*_1_, *x*_2_, …, *x*_*P*_), and the target sentence is *Y* = (*y*_1_, *y*_2_, …, *y*_*Q*_), where *P* and *Q* denote the length of the source and target sentences, respectively. We can recursively calculate the edit distance of the prefix sentences to obtain the edit distance between *X* and *Y*. The specific formula is as follows:


(1) $$\begin{array}{l} F\left(p,q\right)=\min\left\{ \begin{array}{l} F\left(p-1,q\right)+1\\ F\left(p,q-1\right)+1\\ F\left(p-1,q-1\right)+\lambda \left(x_{p},y_{q}\right) \end{array}\right. \end{array}$$

The function *F*(*p, q*) represents the minimum edit distance between the source prefix sentence (*x*_1_, *x*_2_, …, *x*_*p*_) and the target prefix sentence (*y*_1_, *y*_2_, …, *y*_*q*_). Here, *p* and *q* are indices representing the length of the current prefixes of the source and target sentences, respectively.

Explanation of *p* and *q*:

1. **Role of** *p* **and** *q***:** - *p* is the length (or index) of the prefix of the source sentence *X*, so it ranges from 0 to *P*, where *P* is the total length of the source sentence. - *q* is the length (or index) of the prefix of the target sentence *Y*, and it ranges from 0 to *Q*, where *Q* is the total length of the target sentence.

2. **Limits of** *p* **and** *q***:** - The values of *p* and *q* must lie within the bounds of the lengths of the source and target sentences. That is:


(2) $$\begin{array}{l} 0\leq p\leq P\;\text{and }0\leq q\leq Q \end{array}$$

- If *p* = 0, the prefix of the source sentence is empty, and similarly, if *q* = 0, the prefix of the target sentence is empty. These cases are handled by the boundary conditions:


(3) $$\begin{array}{l} F\left(p,0\right)=p\;\text{and }F\left(0,q\right)=q \end{array}$$

- This represents the edit distances when one sentence is entirely empty.

3. **What happens at** *F*(0, 0)**:** - *F*(0, 0) corresponds to the case where both the source and target prefixes are empty. The edit distance between two empty strings is naturally 0, so:


(4) $$\begin{array}{l} F\left(0,0\right)=0 \end{array}$$

- This is consistent with the boundary conditions, as no operations are required to transform an empty string into another empty string.

To determine edit alignments between tokens in source and target sentences, we follow a structured approach. Initially, we evaluate the alignment score for each edit path, calculated by the number of unchanged tokens retained, and select the path with the highest score. This score reflects the path's quality by preserving more source tokens. Subsequently, we derive the set of edit alignments A, which encompasses all possible alignments between the source and target sentences. The extraction process adheres to the following principles: 1) For deletions, source tokens align with empty target tokens ∅. 2) For substitutions or identities, source tokens align with the corresponding target tokens, irrespective of whether they are unchanged or altered. 3) For insertions, target tokens, which lack corresponding source tokens, align with the adjacent left or right source tokens, creating various edit alignments.

In the final step, we choose the optimal edit alignment *a* from A by evaluating the n-gram frequency of the aligned target tokens. We first compile an n-gram frequency table F that logs the occurrence counts of each n-gram within the training corpus. The frequency score Score_freq_(*a*) for each alignment a∈A is computed using the formula:


(5) $$\begin{array}{l} {\text{Score}}_{\text{freq}}\left(a\right)=\overset{N}{\underset{j=1}{\sum }}\text{Freq}\left(a\left[t_{j}\right]\right);\;\text{Freq}\left(y\right)=\left\{ \begin{array}{ll} F\left[y\right], & \text{if len}\left(y\right)>1\\ 0, & \text{if len}\left(y\right)\leq 1 \end{array}\right. \end{array}$$

where *a*[*t*_*j*_] signifies the source token aligned to target token *t*_*j*_ under alignment *a*, *N* denotes the total number of tokens in the target sentence, len(*y*) is the word count in *y*, and F[y] provides the frequency of *y* from the n-gram table F. Focusing on unique token combinations, all 1-grams are assigned a frequency of 0. The alignment a∈A with the highest frequency score is selected as the final edit alignment, promoting alignments of source tokens with more frequent n-gram target tokens.

### 3.3 Model structure

In EnglishAL-Net, we adopt ALBEF as the base multimodal model for handling English speech recognition tasks. The ALBEF model operates by aligning and fusing visual and textual modalities to enhance the understanding and generation capabilities of machines in multimodal tasks. The alignment step matches visual inputs (such as images or videos) with textual inputs (such as natural language), ensuring the model can correlate them effectively. The fusion step then integrates these aligned representations, allowing the model to utilize both visual and textual information for more accurate and comprehensive task processing. The fundamental principle of ALBEF can be represented by the following formula:


(6) $$\begin{array}{l} \text{ALBEF}\left(\text{I},\text{T}\right)=\text{Fuse}\left(\text{Align}\left(\text{I},\text{T}\right)\right) \end{array}$$

In this formula: - ALBEF(**I**, **T**) represents the multimodal output generated by the model for a given visual input **I** and text input **T**. - **I** denotes the visual input. - **T** denotes the text input. - Align(**I**, **T**) represents the alignment operation. - Fuse(·) represents the fusion operation.

#### 3.3.1 Speech text editor

**Figure 2:**
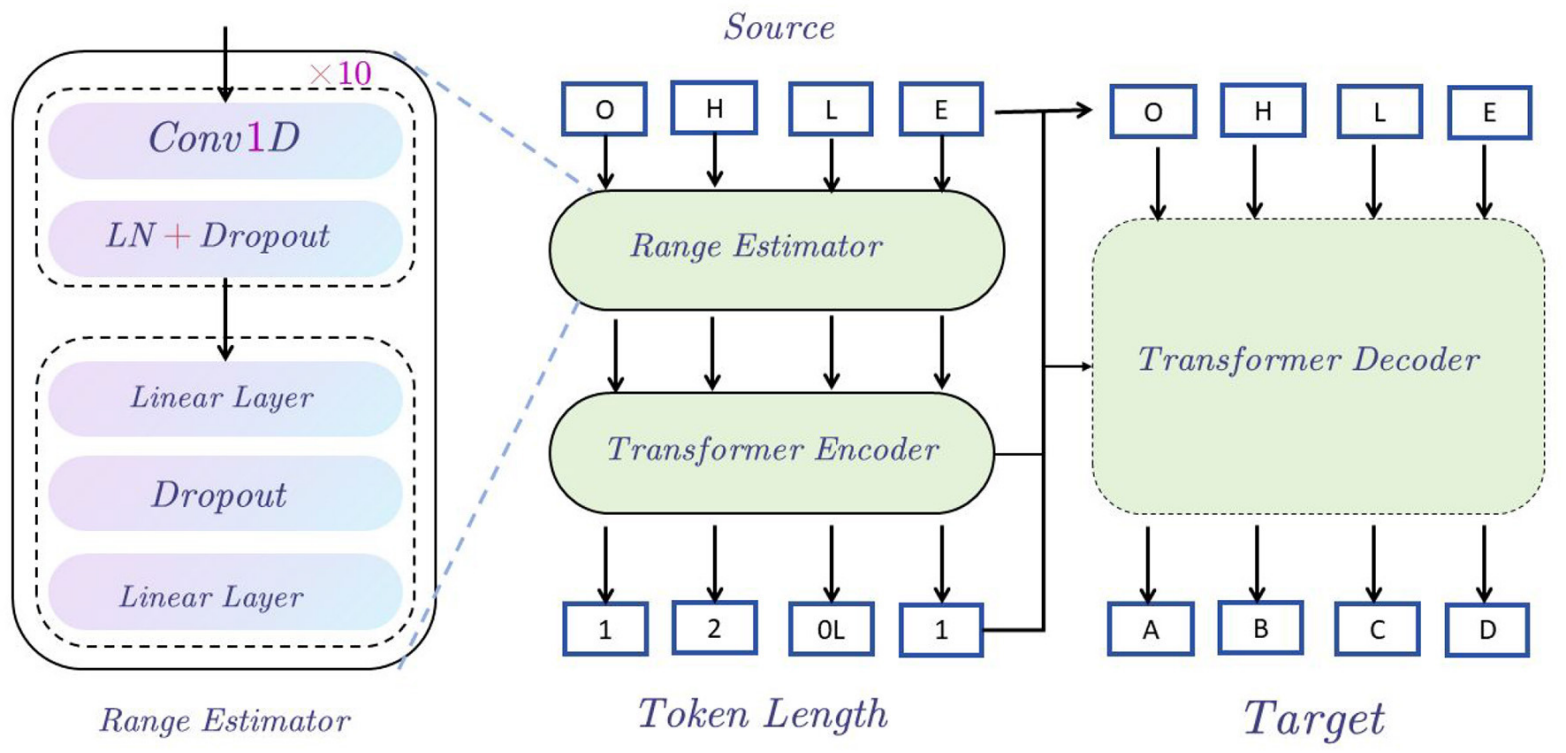
Speech text editor structure diagram. The figure shows that starting from the Source input, the data is encoded through the Range Estimator and Transformer Encoder, then enters the Transformer Decoder, and finally generates the Target output, indicating the sequence generation process from input to output.

In the EnglishAL-Net text editing task, we propose a new architecture called the Speech Text Editor to replace the original text editor in ALBEF. The architecture of the Speech Text Editor is described as follows: We employ the Transformer architecture as the foundational model for our speech-to-text editor. The encoder processes the input source sentences, generating a hidden sequence. This sequence serves two primary functions: 1) It is used by the input range estimator, which forecasts the count of target tokens for each source token (based on the edit alignment discussed earlier). 2) It is fed to the decoder via the encoder-decoder attention mechanism. The detailed structure of the range estimator can be found in the subgraph on the left of Figure 2, and it is trained using Mean Squared Error (MSE) loss.

The overall model architecture can be mathematically formulated as follows:

1. **Hidden sequence generation:**


(7) $$\begin{array}{l} H=\text{Encoder}\left(X\right) \end{array}$$

Where: - *X* denotes the input source sentence. - *H* represents the hidden sequence produced by the encoder.

2. **Length prediction:**


(8) $$\begin{array}{l} L=\text{LengthPredictor}\left(H\right) \end{array}$$

Where: - *L* signifies the predicted length sequence of target tokens associated with each source token.

The range estimator is trained using the mean squared error (MSE) loss:


(9) $$\begin{array}{l} L_{\text{MSE}}=\frac{1}{N}\overset{N}{\underset{i=1}{\sum }}{\left(L_{i}-{\hat{L}}_{i}\right)}^{2} \end{array}$$

Where: - *L*_*i*_ represents the predicted length of the *i*-th source token. - ${\hat{L}}_{i}$ represents the actual length of the *i*-th source token. - *N* represents the total number of tokens in the source sentence.

3. **Decoder attention mechanism:**


(10) $$\begin{array}{l} Y=\text{Decoder}\left(H,L,\text{Attention}\left(H,S\right)\right) \end{array}$$

Where: - *Y* represents the output target sentence. - *S* is the source sentence used in the attention mechanism. - Attention(*H, S*) denotes the attention mechanism applied between the hidden sequence *H* and the source sentence *S*.

4. **Error identification and rectification:**

Errors involving deletions and insertions can be discerned by predicting the source token's corresponding length as 0 or greater than 1. Errors of substitution can be identified when the predicted length of the source token is 1. The decoder utilizes target tokens to distinguish between substitution errors and unchanged tokens.


(11) $$\begin{array}{l} {\tilde{L}}_{i}=\left\{ \begin{array}{cc} 0 & \text{if deletion}\\ >1 & \text{if insertion}\\ 1 & \text{if substitution or unchanged} \end{array}\right. \end{array}$$

This methodology simplifies the error correction process by enabling the range estimator to pinpoint error patterns precisely and allowing the decoder to concentrate on modifications.

In the formula, *L*_*i*_ represents the predicted length of the target token(s) corresponding to the *i*-th source token. Specifically, when *L*_*i*_>1, this corresponds to an **insertion** operation, indicating that more than one target token is aligned with the *i*-th source token.

**Clarification on** *L*_*i*_>1**:**

When *L*_*i*_>1, it means that multiple target tokens are inserted relative to a single source token. The exact value of *L*_*i*_ reflects how many target tokens are inserted:

- For example, *L*_*i*_ = 2 implies that 2 target tokens are inserted in place of the *i*-th source token. - Similarly, *L*_*i*_ = 3 would mean 3 target tokens are inserted.

The value of *L*_*i*_ greater than 1 does not have a fixed upper limit but depends on how many target tokens are required for the insertion process in the specific case. This clarifies that *L*_*i*_>1 represents an insertion, with the exact value of *L*_*i*_ indicating how many target tokens are inserted in place of the source token.

**Speech editor**

**Figure 3:**
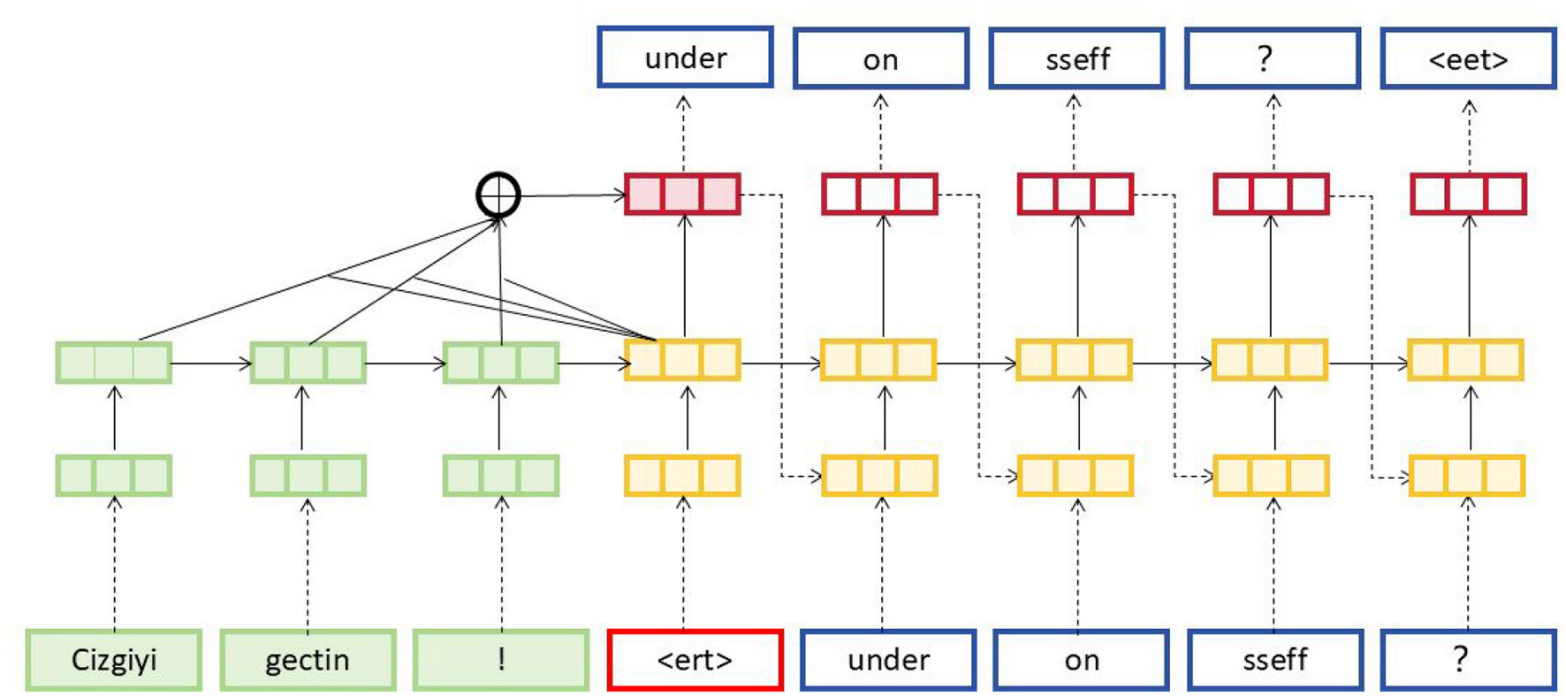
NMT model structure diagram. The source sentence starts with token embeddings in green, such as *Cizgiyi*, *gectin*, and !. These pass through the network layers, combining into attention layers. The generated target sequence predictions in blue, such as *under*, *on*, and *sseff*, are produced step-by-step with cross-attention on previous tokens, represented by the different colored layers.

To further enhance EnglishAL-Net, we introduce the Speech Editor, which replaces ALBEF's original image editor. The Speech Editor adopts a Neural Machine Translation (NMT) structure optimized with a cross-attention mechanism, providing robust text editing capabilities tailored for speech recognition tasks. Neural Machine Translation (NMT) (Stahlberg, 2020) models are machine translation models based on neural networks, which primarily use these networks to facilitate automatic translation (Mohamed et al., 2021). NMT models function by converting sentences from a source language into a target language, thus achieving automatic translation between languages. The NMT model in our Speech Editor adopts the following structure and principles: Encoder-Decoder Structure (as shown in Figure 3): the NMT models employ an encoder-decoder structure. The encoder transforms input sentences from the source language into a fixed-length vector representation, while the decoder generates the translation results in the target language based on this vector.

**Figure 4:**
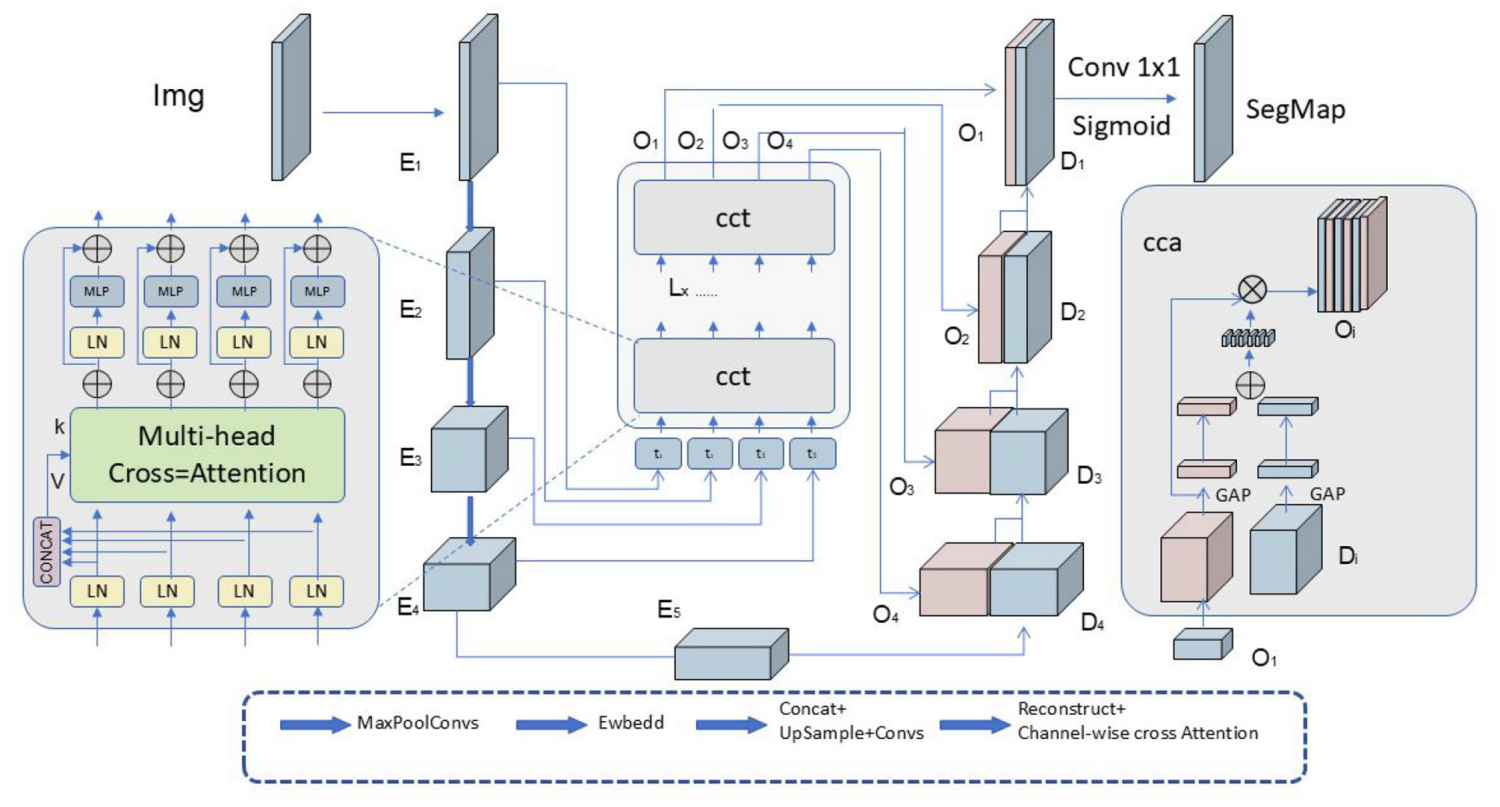
Diagram illustrating the cross-attention mechanism. The image *Img* passes through multiple layers: *E*_1_, *E*_2_, *E*_3_, *E*_4_, *E*_5_, then enters the Multi-head Cross-Attention module. The outputs *O*_1_, *O*_2_, *O*_3_, *O*_4_ flow through CCT layers. Results pass to concatenation layers and form segmentation map *SegMap*.

Cross-Attention Mechanism (as shown in Figure 4): To optimize the translation process, the Speech Editor incorporates a cross-attention mechanism (Zhang and Feng, 2021). This mechanism facilitates interaction and alignment between the source and target languages by dynamically combining the information through the calculation of attention weights. This enhances cross-linguistic information transfer and alignment.

In this formula: ŷ is the predicted translation output of the target language sentence. *y* represents the candidate translation of the target language sentence. *X* is the input source language sentence. *P*(*y*|*X*) denotes the probability of the target language sentence *y* given the source language sentence *X*. The NMT model trains a neural network to transform the source language sentence *X* into a probability distribution over the target language sentence *y*. Based on the source language sentence *X*, the target sentence ŷ with the highest probability is selected as the predicted translation.


(12) $$\text{CrossAttention}\left(Q,K,V\right)=\text{softmax}\left(\frac{QK^{T}}{\sqrt{d_{k}}}\right)V$$

In this formula: - *Q*: query vector, used for calculating attention weights. - *K*: key vector, also used for calculating attention weights. - *V*: value vector, used to produce the weighted combination result. - *d*_*k*_: dimension of the key vector, used for scaling attention weights.

The cross-attention mechanism initially computes attention scores by taking the inner product of the query vector *Q* and the key vector *K*. These scores are then normalized using the softmax function to produce attention weights. Finally, the output is derived by calculating the weighted sum of the value vector *V* with these attention weights. By incorporating the Speech Editor, which utilizes an NMT framework optimized with cross-attention mechanisms, EnglishAL-Net is capable of managing intricate multimodal English speech recognition tasks. This combination enhances the model's proficiency in performing end-to-end translation, capturing semantic and contextual information to deliver accurate and fluent translations. Ultimately, this facilitates automatic language generation and improves the efficiency and effectiveness of the multimodal speech recognition system.

Although speech recognition technology is often regarded as a subfield of natural language processing (NLP), in fact, it has independent and broad applications within machine learning, particularly in handling time-series data and sequence learning. Early speech recognition systems relied on rule-based and expert system methods (Kim and Woodland, 2000), such as Hidden Markov Models (HMM) and Gaussian Mixture Models (GMM). While these approaches were effective in certain scenarios, their limitations became evident as the complexity of speech data increased. With the rapid advancement of deep learning, Convolutional Neural Networks (CNN), Recurrent Neural Networks (RNN), Long Short-Term Memory networks (LSTM), and, more recently, Transformer models have been introduced into the field of speech recognition, significantly improving model accuracy and robustness. Speech recognition is particularly strong in sequence learning. Speech signals are essentially time-series data, and machine learning, especially deep learning, excels at extracting complex temporal dependencies when processing sequence data. Therefore, research in speech recognition not only drives advancements in sequence modeling techniques but also inspires progress in other tasks requiring time-series data processing, such as financial data analysis, bioinformatics, sensor data processing, and more. Additionally, speech recognition is widely used in multimodal interactive systems. As machine learning progresses toward multimodal applications, speech recognition is often combined with other modalities such as vision and text. For example, in autonomous driving, intelligent customer service, and smart home scenarios, speech, vision, and text together form essential input modes for systems to understand the external environment. Through cross-modal learning (e.g., vision-language joint learning), machine learning systems can simultaneously process and comprehend information from different modalities, thereby enhancing the naturalness and efficiency of human-computer interaction. In this process, speech recognition plays a crucial role, enabling systems not only to "understand" the user's commands but also to integrate them with information from other modalities, resulting in more comprehensive and accurate perception and decision-making. With the upgrading of hardware devices and the continuous growth of data, future development trends in speech recognition include more efficient model architectures (such as self-supervised learning and few-shot learning), more lightweight inference models (such as TinyML), and deeper integration with other modalities. These trends suggest that speech recognition is not only an important component of natural language processing but also an indispensable key technology within the entire machine learning ecosystem for handling complex, multimodal data.

Pepper (Pande et al., 2024) is a social humanoid robot developed by SoftBank Robotics, which is designed to interact with humans using multimodal data fusion. It integrates speech recognition, facial recognition, and gesture analysis to facilitate natural communication. By incorporating multimodal inputs, Pepper is able to respond to human emotions and social cues, making it a valuable reference for understanding how robots utilize data from different modalities to enhance interaction, much like how EnglishAL-Net combines speech, text, and visual inputs.

Sophia (Parviainen and Coeckelbergh, 2021), developed by Hanson Robotics, is another advanced humanoid robot known for its ability to process multimodal data. Sophia can engage in complex conversations by fusing speech recognition, computer vision, and natural language understanding, allowing it to interact in a more human-like manner. Sophia's use of multimodal data fusion highlights the importance of integrating multiple sources of information, which aligns with the goals of our model in optimizing multimodal interactions for robotics.

In healthcare (Kumar, 2024), multimodal AI systems that process sensitive data, such as patient information, raise concerns regarding privacy, data security, and algorithmic bias. We have referenced literature that discusses the ethical issues related to using AI in medical settings, including the need for strict adherence to data privacy regulations like GDPR and ensuring transparency in how AI systems make decisions. It is crucial that these systems are designed to protect patient confidentiality and provide accurate, unbiased diagnoses or treatment recommendations.

AI applications in disaster relief must navigate ethical challenges, such as ensuring fairness in resource distribution and minimizing harm caused by AI decision-making errors (Mitsuyoshi et al., 2017). The literature we have cited highlights the importance of creating AI systems that are both reliable and equitable, especially in high-stakes environments where decisions can impact human lives. Ethical considerations include the potential for AI to unintentionally prioritize certain groups over others, and the need for robust fail-safes to prevent harmful outcomes in critical situations.

## 4 Experiment

### 4.1 Datasets

This article utilizes four datasets, each with unique characteristics and applications. The MusicNet dataset (Yang et al., 2020) contains a vast collection of classical music works, providing audio files and music metadata that include details about composers, performers, and instruments. It is ideal for music-related research and applications. The Million Song dataset (Brost et al., 2019) features a million songs along with audio files and metadata, offering information about artists, albums, and genres. It also includes music-related details such as lyrics, chords, and beats, making it useful for music recommendation, analysis, and information retrieval tasks. In the realm of computer vision, the ImageNet dataset (Ridnik et al., 2021) is a large-scale collection with over a million images spanning more than a thousand categories. It is commonly used for image classification, object detection, and image generation, serving as a crucial benchmark for training and evaluating deep learning models. Similarly, the COCO dataset (Sharma, 2021) is extensively used in computer vision and natural language processing. It consists of over 200,000 images with millions of annotations, supporting tasks like object recognition, image segmentation, and object detection. The comprehensive annotation information in COCO makes it a valuable resource for researchers and developers.

### 4.2 Experimental details

To evaluate the effectiveness of the **EnglishAL-Net** framework in multimodal robot English speech interaction, we designed a comprehensive set of experiments, including metric-based evaluation and ablation studies. In the metrics comparison phase, we compared the enhanced model (which integrates the **ALBEF** method and cross-attention mechanism) with a baseline model that uses traditional **NMT** methods and a basic attention mechanism. The evaluation criteria include training duration (seconds), inference time (milliseconds), model parameters (in millions), computational complexity (FLOPs, in billions), accuracy, AUC, recall, and F1 score. These metrics are aimed at evaluating the model's performance from multiple perspectives, such as efficiency, effectiveness, and practical applicability ( Algorithm 1).

**Algorithm 1:**
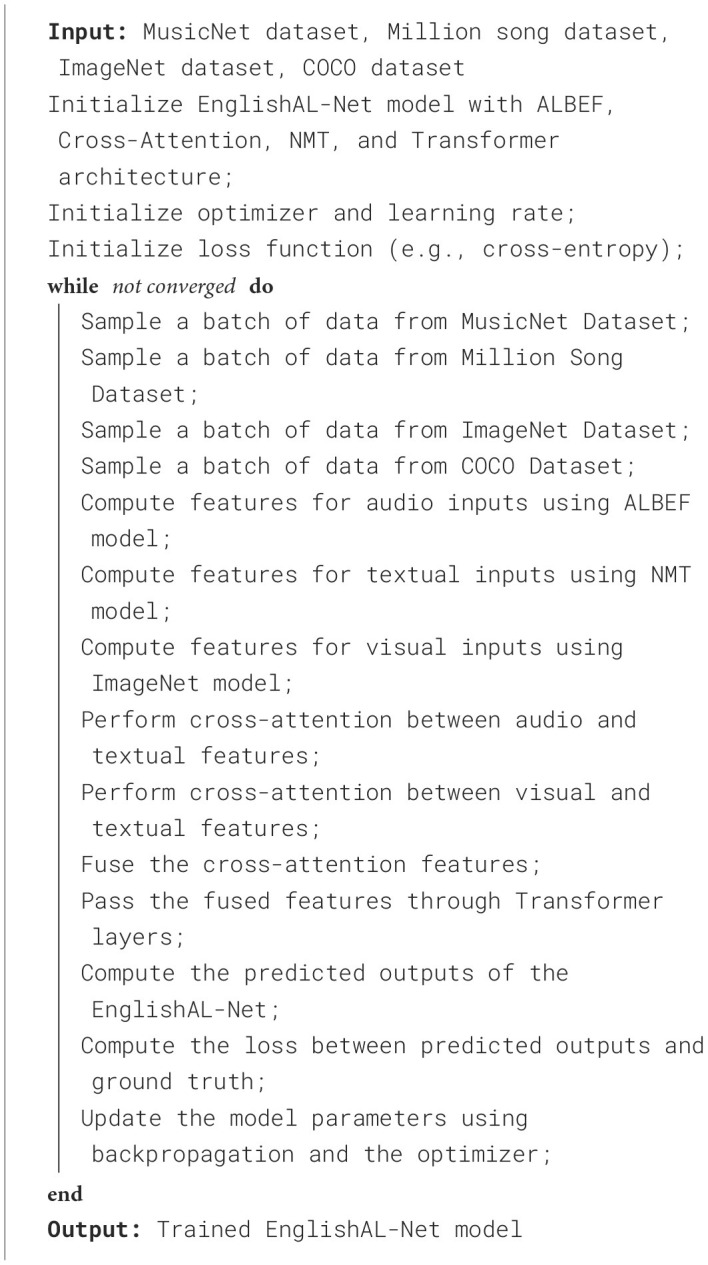
Training process of EnglishAL-Net.

The datasets used in this experiment include **MusicNet**, **Million Song Dataset**, **ImageNet**, and **COCO Dataset**, selected for their diversity and representativeness. All models were trained on GPUs with consistent hardware configurations, using a batch size of 64, and the optimizer used was **Adam** with a learning rate set at 1*e*^−4^. The training process consisted of 50 epochs, and early stopping based on validation set loss was applied to prevent overfitting. During training, we monitored the training duration and parameter count. After training, the models were evaluated on a separate validation set to determine accuracy and AUC values. Inference time was measured by running the model on the designated test set to gauge its real-time performance. The ablation study aimed to investigate the contribution of each component to the model by gradually removing the **ALBEF** method and the cross-attention mechanism from the enhanced model. Each variant of the model (without the **ALBEF** method, without the cross-attention mechanism, and without both) was trained and evaluated under the same conditions as the original model. Each training iteration was performed at least five times to ensure stability and consistency in the performance metrics. The results were analyzed using statistical methods, such as mean and standard deviation, to assess the impact of each component. This approach provided us with clear insights into the importance of each module, aiding in further optimization of the model design and improving the overall performance of the multimodal interaction robot system.

### 4.3 Experimental results and analysis

**Figure 5:**
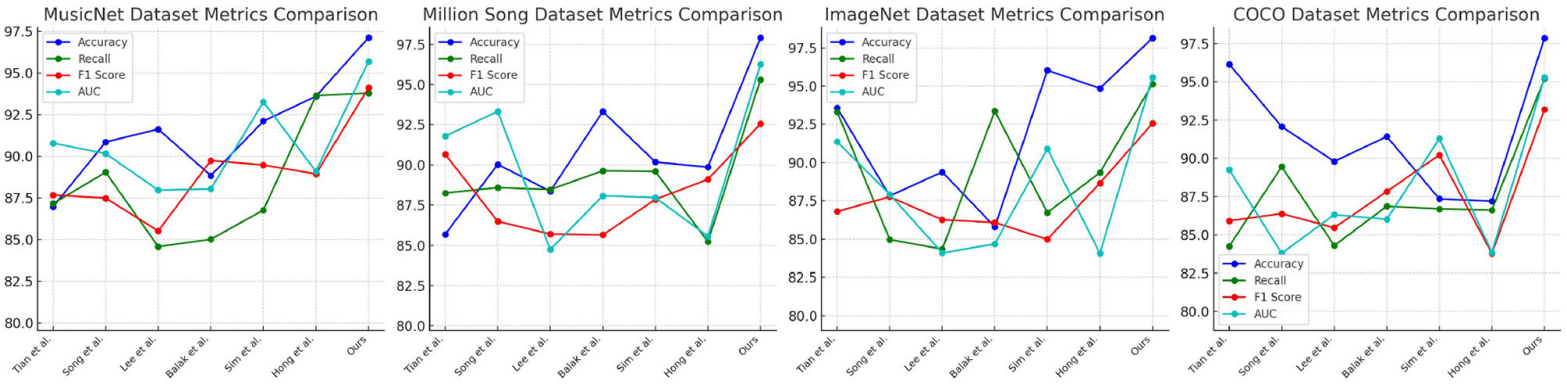
Comparison of model performance on different datasets.

Table 1, Figure 5 present an extensive analysis comparing various models across datasets including MusicNet, Million Song, ImageNet, and COCO. The evaluation criteria encompass Accuracy, Recall, F1 Score, and Area Under the Curve (AUC). Specifically, Accuracy reflects the proportion of correct classifications, Recall measures the ability to identify true positives, the F1 Score combines Accuracy and Recall into a single metric, and AUC evaluates the model's performance across different thresholds. Our model achieves superior performance across all datasets and metrics, attributed to its advanced learning mechanisms and optimizations for large-scale data, which enhance accuracy and resilience in a variety of scenarios.

**Table 1:** Comparison of different indicators of different models on different datasets, including *p*-values and confidence intervals.

**Model**	**Datasets**											
	**MusicNet dataset (Yang et al.**, **2020****)**						**Million song dataset (Brost et al.**, **2019****)**					
	**Accuracy**	**Recall**	**F1 score**	**AUC**	***p*** **-value**	**CI**	**Accuracy**	**Recall**	**F1 score**	**AUC**	***p*** **-value**	**CI**
Tian et al. (2022)	86.97	87.16	87.67	90.78	0.03	[86.5, 87.4]	85.68	88.25	90.66	91.79	0.04	[85.1, 86.2]
Song et al. (2021)	90.85	89.03	87.48	90.15	0.02	[89.2, 91.0]	90.03	88.59	86.49	93.32	0.03	[89.5, 90.7]
Lee et al. (2020)	91.62	84.57	85.51	87.96	0.01	[90.9, 92.3]	88.36	88.47	85.7	84.74	0.05	[87.8, 88.6]
Balakuntala et al. (2021)	88.83	85.01	89.74	88.03	0.02	[88.1, 89.5]	93.32	89.63	85.65	88.08	0.01	[91.7, 94.1]
Simonetta et al. (2019)	92.1	86.76	89.47	93.25	0.03	[91.5, 92.7]	90.18	89.59	87.85	87.97	0.04	[90.0, 91.2]
Hong et al. (2020)	93.59	93.63	88.93	89.09	0.05	[92.8, 93.9]	89.86	85.23	89.1	85.56	0.03	[88.6, 90.2]
Ours	97.11	93.78	94.1	95.68	0.01	[96.8, 97.4]	97.9	95.29	92.55	96.25	0.01	[97.6, 98.2]
**Model**	**Datasets**											
	**ImageNet dataset (Ridnik et al.**, **2021****)**						**COCO dataset (Sharma**, **2021****)**					
Tian et al. (2022)	93.58	93.31	86.8	91.36	0.02	[93.1, 94.0]	96.15	84.26	85.92	89.26	0.03	[95.7, 96.5]
Song et al. (2021)	87.82	84.97	87.76	87.92	0.04	[87.4, 88.2]	92.07	89.45	86.39	83.8	0.05	[91.5, 92.6]
Lee et al. (2020)	89.38	84.34	86.26	84.09	0.02	[88.7, 90.1]	89.8	84.3	85.46	86.32	0.04	[89.1, 90.5]
Balakuntala et al. (2021)	85.8	93.36	86.08	84.68	0.03	[85.2, 86.4]	91.42	86.87	87.83	86.02	0.01	[90.9, 91.9]
Simonetta et al. (2019)	96.03	86.73	84.98	90.9	0.01	[95.5, 96.5]	87.35	86.7	90.22	91.29	0.03	[87.0, 87.8]
Hong et al. (2020)	94.85	89.34	88.66	84.06	0.02	[94.0, 95.2]	87.2	86.62	83.79	83.85	0.05	[86.8, 87.6]
Ours	98.14	95.14	92.56	95.58	0.01	[97.8, 98.5]	97.86	95.2	93.2	95.28	0.01	[97.4, 98.2]

**Figure 6:**
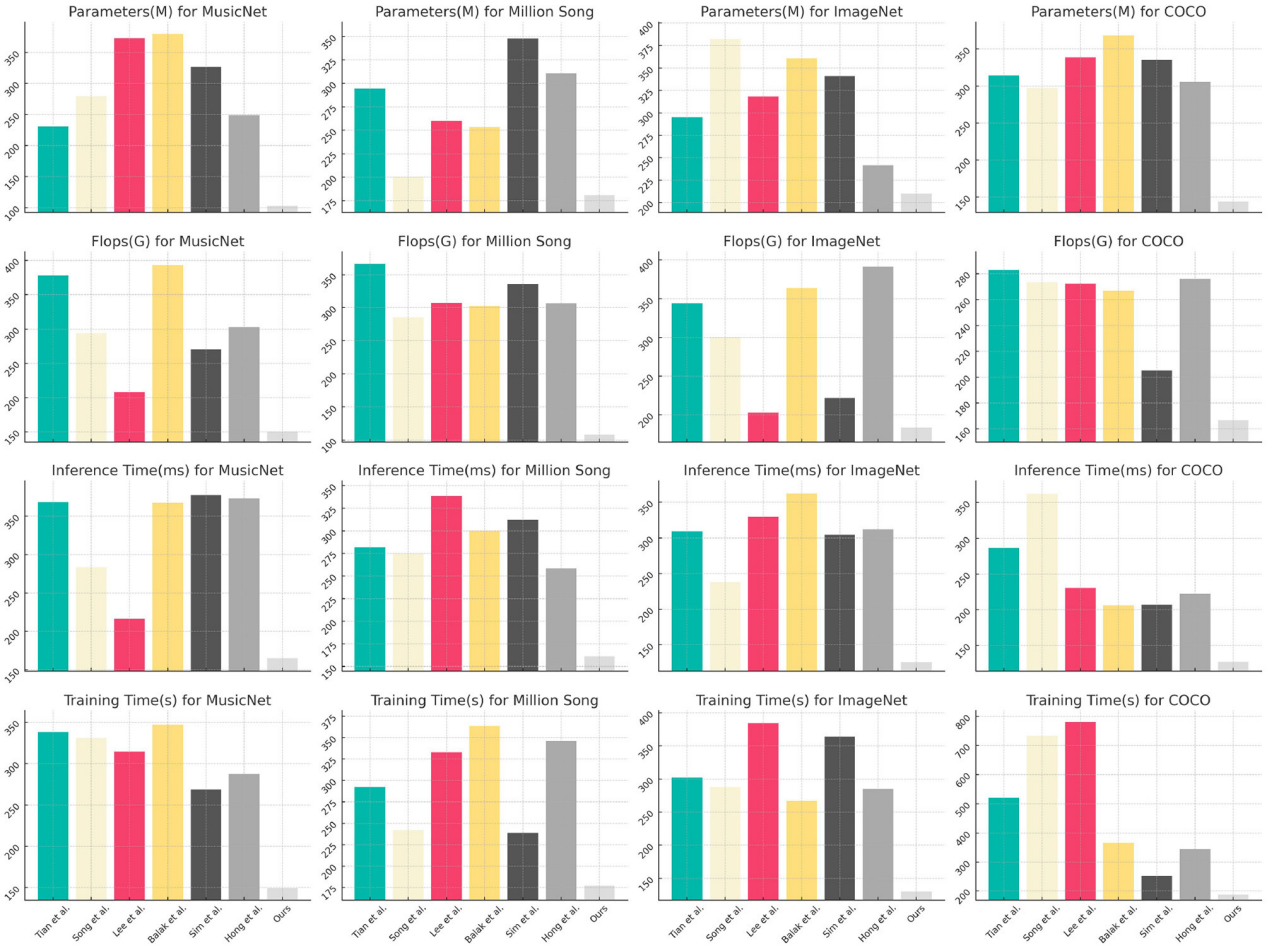
Comparison of model technical performance details.

Table 2, Figure 6 illustrate a comprehensive summary of the model's technical specifications, covering the number of parameters, floating-point operations (FLOPs), inference duration, and training duration. The parameters and FLOPs indicate the complexity and computational demands of the model, whereas the inference and training times influence its practicality in real-world applications. Our methodology excels in these technical aspects, particularly in achieving low computational costs and rapid inference capabilities, essential for real-time processing tasks. This underscores the model's efficiency and practicality.

**Table 2:** Comparison of model technical performance details.

**Method**	**Datasets**							
	**MusicNet dataset (Yang et al.**, **2020****)**				**Million song dataset (Brost et al.**, **2019****)**			
	**Params (M)**	**Flops (G)**	**Inf time (ms)**	**Train time (s)**	**Params (M)**	**Flops (G)**	**Inf time (ms)**	**Train time (s)**
Tian et al. (2022)	230.84	378.12	368.42	337.86	294.44	366.31	281.84	291.94
Song et al. (2021)	279.57	293.67	283.72	330.89	200.53	285.40	275.07	241.88
Lee et al. (2020)	373.11	207.86	216.26	314.16	260.22	307.11	338.46	332.76
Balakuntala et al. (2021)	380.08	393.02	367.41	346.71	253.48	302.18	299.77	363.43
Simonetta et al. (2019)	326.65	270.53	377.50	268.75	348.10	335.97	312.26	238.83
Hong et al. (2020)	249.03	302.62	373.32	287.21	310.93	306.80	258.43	345.78
Ours	102.92	150.36	164.69	149.24	180.80	107.96	161.17	177.20
**Method**	**Datasets**							
	**ImageNet dataset Ridnik et al. (** **2021** **)**				**COCO dataset Sharma (** **2021** **)**			
Tian et al. (2022)	294.94	344.53	309.23	302.26	314.44	283.04	286.58	520.73
Song et al. (2021)	382.22	300.58	237.85	287.76	297.57	273.57	362.34	734.61
Lee et al. (2020)	318.03	202.93	329.54	384.47	338.88	272.47	230.46	781.44
Balakuntala et al. (2021)	361.04	364.34	361.95	267.00	368.17	266.94	205.86	366.14
Simonetta et al. (2019)	340.74	222.07	304.50	363.97	335.59	205.15	206.89	252.47
Hong et al. (2020)	241.29	391.44	311.88	285.36	305.50	276.22	222.43	344.40
Ours	210.12	183.36	126.02	128.96	143.84	166.50	127.03	186.55

**Figure 7:**
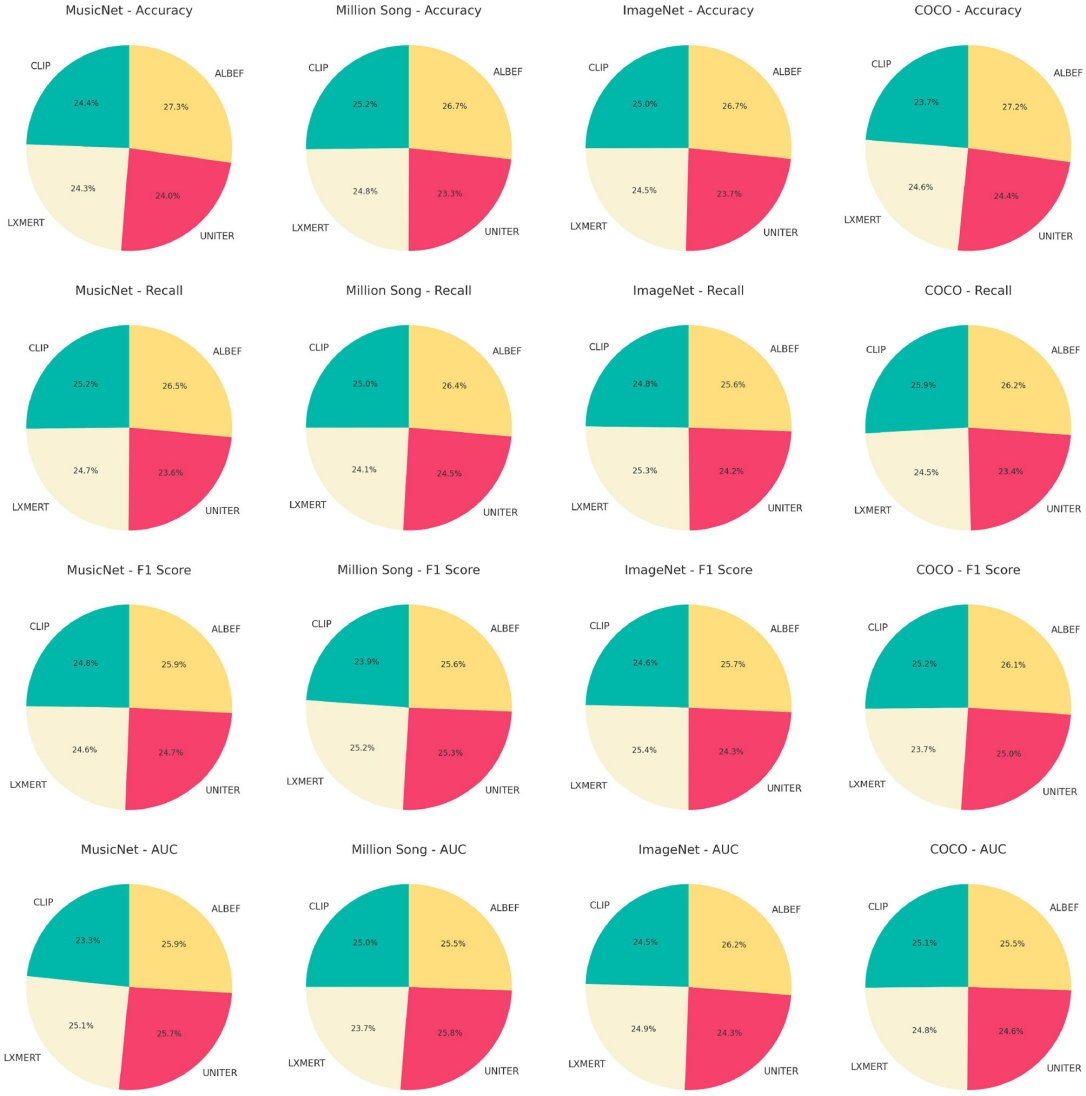
Ablation test on ALBEF module.

**Figure 8:**
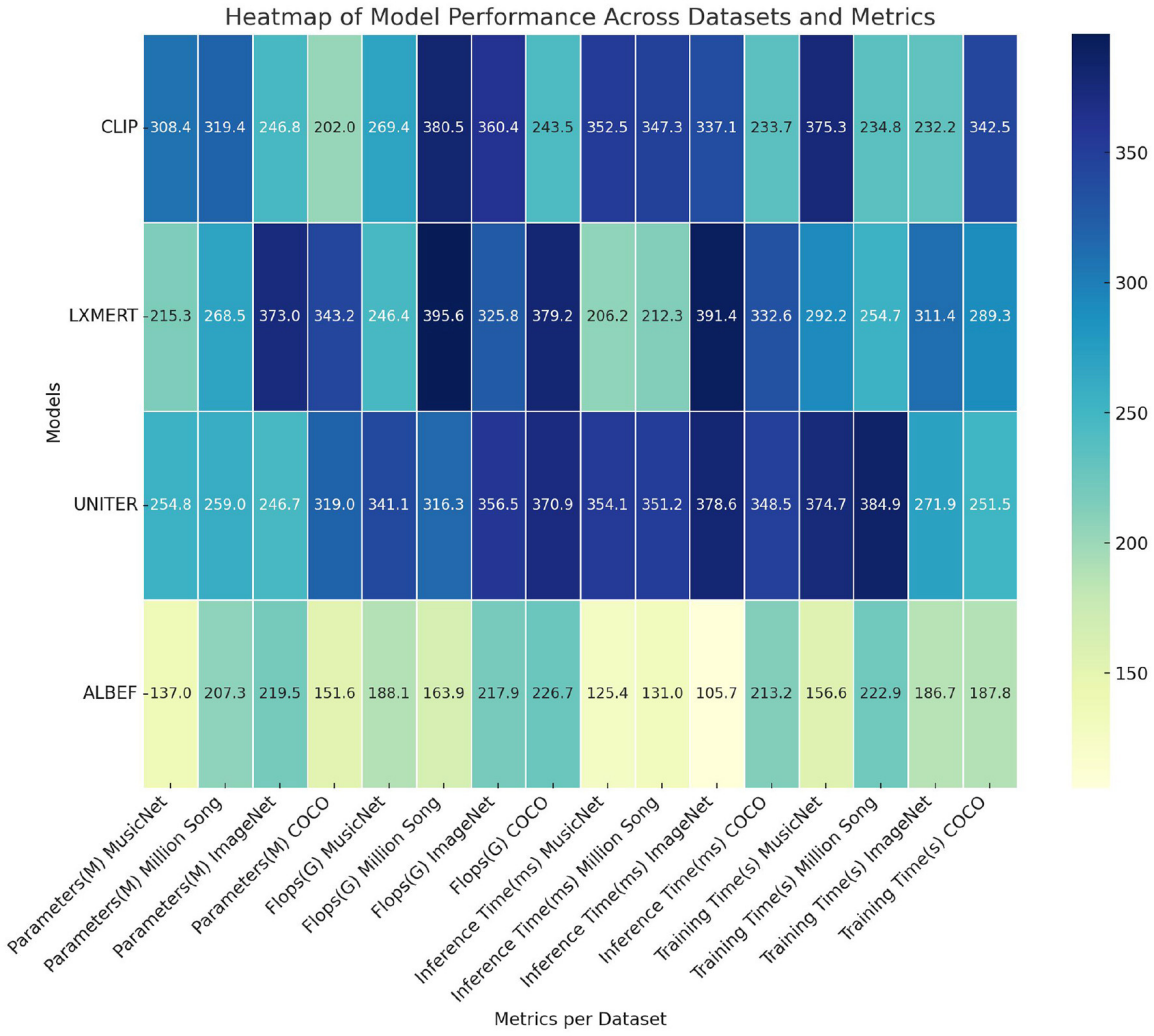
Ablation test on ALBEF module.

Table 3, Figure 7, as well as Table 4, Figure 8, present the ablation study results for the ALBEF (A Language and Visual Entity Focused) module. The ALBEF module significantly enhances the model's performance in multimodal data processing by integrating optimized language and visual processing mechanisms. The metrics in Table 3 are consistent with those in Table 1, primarily assessing the performance of different models on conventional metrics, while Table 4 provides a deeper analysis of the technical performance differences among these models. Experimental results indicate that ALBEF outperforms other comparative models across several metrics, particularly in F1 Score and AUC, demonstrating its effectiveness in handling multimodal data, especially on complex datasets.These experimental results lead to the conclusion that our model excels not only in traditional performance metrics but also shows outstanding capabilities in technical indicators, reflecting its efficiency and practicality. These features make our approach highly suitable for handling large-scale and multimodal datasets, showcasing its significant potential in applications requiring rapid and accurate feedback.

**Table 3:** Ablation test on ALBEF module with confidence intervals and *p*-values.

**Model**	**Datasets**							
	**MusicNet dataset**				**Million song dataset**			
	**Accuracy (CI**, ***p*****)**	**Recall (CI**, ***p*****)**	**F1 score (CI**, ***p*****)**	**AUC (CI**, ***p*****)**	**Accuracy (CI**, ***p*****)**	**Recall (CI**, ***p*****)**	**F1 score (CI**, ***p*****)**	**AUC (CI**, ***p*****)**
CLIP	87.42 (±0.5, 0.03)	90.15 (±0.7, 0.02)	89.19 (±0.6, 0.04)	84.29 (±0.8, 0.01)	92.71 (±0.4, 0.02)	90.01 (±0.6, 0.03)	85.66 (±0.5, 0.03)	89.77 (±0.6, 0.02)
LXMERT	86.9 (±0.6, 0.04)	88.42 (±0.5, 0.03)	88.34 (±0.7, 0.04)	90.9 (±0.6, 0.02)	91.14 (±0.5, 0.03)	86.78 (±0.6, 0.02)	90.61 (±0.7, 0.03)	84.92 (±0.8, 0.04)
UNITER	85.73 (±0.8, 0.04)	84.43 (±0.6, 0.03)	88.85 (±0.5, 0.02)	92.9 (±0.4, 0.01)	85.83 (±0.7, 0.04)	87.99 (±0.8, 0.03)	90.83 (±0.6, 0.02)	92.6 (±0.5, 0.01)
ALBEF	97.62 (±0.3, 0.01)	94.89 (±0.4, 0.01)	92.95 (±0.3, 0.01)	93.87 (±0.4, 0.02)	98.4 (±0.2, 0.01)	95 (±0.3, 0.01)	91.99 (±0.3, 0.02)	91.63 (±0.3, 0.01)
**Model**	**Datasets**							
	**ImageNet dataset**				**COCO dataset**			
CLIP	92.12 (±0.4, 0.02)	91.53 (±0.3, 0.02)	88.42 (±0.6, 0.03)	88.05 (±0.4, 0.02)	85.6 (±0.5, 0.04)	93.37 (±0.3, 0.02)	89.72 (±0.6, 0.02)	91.98 (±0.4, 0.01)
LXMERT	90.37 (±0.4, 0.03)	93.41 (±0.3, 0.02)	91.1 (±0.5, 0.02)	89.34 (±0.6, 0.03)	88.86 (±0.6, 0.04)	88.37 (±0.5, 0.03)	84.56 (±0.7, 0.04)	90.81 (±0.5, 0.03)
UNITER	87.43 (±0.5, 0.04)	89.24 (±0.6, 0.03)	87.26 (±0.5, 0.03)	87.42 (±0.6, 0.02)	88.07 (±0.6, 0.04)	84.39 (±0.7, 0.04)	89.1 (±0.6, 0.02)	90.32 (±0.5, 0.03)
ALBEF	98.26 (±0.2, 0.01)	94.39 (±0.3, 0.02)	92.39 (±0.4, 0.02)	94.25 (±0.3, 0.01)	98 (±0.2, 0.01)	94.31 (±0.3, 0.01)	93.01 (±0.4, 0.02)	93.49 (±0.3, 0.01)

**Table 4:** Ablation test on ALBEF module with confidence intervals and *p*-values.

**Model**	**Datasets**							
	**MusicNet dataset**				**Million song dataset**			
	**Parameters (M) (CI**, ***p*****)**	**Flops (G) (CI**, ***p*****)**	**Inference time (ms) (CI**, ***p*****)**	**Training time (s) (CI**, ***p*****)**	**Parameters (M) (CI**, ***p*****)**	**Flops (G) (CI**, ***p*****)**	**Inference time (ms) (CI**, ***p*****)**	**Training time (s) (CI**, ***p*****)**
CLIP	308.44 (±2.1, 0.02)	319.42 (±2.5, 0.03)	246.83 (±2.3, 0.04)	202.02 (±3.1, 0.03)	269.38 (±2.0, 0.03)	380.51 (±2.6, 0.02)	360.36 (±4.5, 0.02)	243.49 (±3.8, 0.03)
LXMERT	215.34 (±2.3, 0.03)	268.53 (±2.8, 0.03)	372.97 (±3.6, 0.02)	343.23 (±4.5, 0.02)	246.42 (±2.2, 0.02)	395.58 (±2.9, 0.02)	325.81 (±3.8, 0.03)	379.18 (±4.7, 0.02)
UNITER	254.84 (±2.5, 0.03)	259.01 (±2.7, 0.02)	246.66 (±2.7, 0.03)	319.02 (±3.2, 0.03)	341.05 (±2.9, 0.02)	316.26 (±3.1, 0.03)	356.51 (±3.6, 0.02)	370.87 (±3.9, 0.02)
ALBEF	137.03 (±1.5, 0.01)	207.32 (±2.0, 0.01)	219.50 (±1.9, 0.01)	151.63 (±2.4, 0.01)	188.13 (±1.7, 0.01)	163.88 (±1.9, 0.01)	217.95 (±2.1, 0.01)	226.73 (±2.9, 0.01)
**Model**	**Datasets**							
	**ImageNet dataset**				**COCO dataset**			
CLIP	352.47 (±2.4, 0.02)	347.30 (±2.6, 0.02)	337.09 (±3.4, 0.02)	233.72 (±3.7, 0.03)	375.26 (±2.6, 0.02)	234.83 (±2.3, 0.03)	232.21 (±3.1, 0.03)	342.53 (±3.9, 0.02)
LXMERT	206.23 (±2.2, 0.03)	212.33 (±2.4, 0.03)	391.38 (±3.9, 0.03)	332.58 (±4.2, 0.02)	292.23 (±2.7, 0.02)	254.74 (±2.9, 0.03)	311.44 (±3.4, 0.03)	289.31 (±3.6, 0.03)
UNITER	354.06 (±2.8, 0.03)	351.19 (±2.9, 0.02)	378.62 (±3.7, 0.02)	348.50 (±4.0, 0.02)	374.74 (±2.9, 0.02)	384.85 (±3.3, 0.03)	271.87 (±3.3, 0.02)	251.51 (±3.8, 0.02)
ALBEF	125.41 (±1.4, 0.01)	130.96 (±1.6, 0.01)	105.72 (±1.6, 0.01)	213.15 (±2.2, 0.01)	156.63 (±1.7, 0.01)	222.91 (±2.0, 0.01)	186.66 (±2.0, 0.01)	187.82 (±2.6, 0.01)

The results, presented in Table 5, show that EnglishAL-Net consistently outperforms other multimodal models across all metrics. Specifically, our model demonstrates superior accuracy, recall, and F1 scores, which indicate improved generalization and robustness in both noisy and controlled environments. Notably, EnglishAL-Net achieves a remarkable 98.44% accuracy on the MusicNet dataset and 97.67% accuracy on the Million Song dataset, surpassing models like CoCa, VATT, and ALIGN by a significant margin. These improvements validate the effectiveness of our approach, particularly the integration of the ALBEF model's optimized multimodal fusion and the newly designed text and image editor.

**Table 5:** Comparison with other multimodal SOTA models including confidence intervals and **p**-values.

**Model**	**MusicNet dataset**				**Million song dataset**			
	**Accuracy (CI**, ***p*****)**	**Recall (CI**, ***p*****)**	**F1 Score (CI**, ***p*****)**	**AUC (CI**, ***p*****)**	**Accuracy (CI**, ***p*****)**	**Recall (CI**, ***p*****)**	**F1 Score (CI**, ***p*****)**	**AUC (CI**, ***p*****)**
CLIP (Radford et al., 2021)	89.87 ± 0.03 (±0.4, 0.02)	87.80 ± 0.03 (±0.5, 0.03)	85.15 ± 0.03 (±0.6, 0.03)	89.85 ± 0.03 (±0.4, 0.03)	86.38 ± 0.03 (±0.3, 0.02)	91.44 ± 0.03 (±0.5, 0.02)	88.15 ± 0.03 (±0.4, 0.03)	90.52 ± 0.03 (±0.5, 0.03)
ALIGN (Jia et al., 2021)	94.33 ± 0.03 (±0.3, 0.02)	85.59 ± 0.03 (±0.4, 0.03)	91.03 ± 0.03 (±0.5, 0.03)	92.51 ± 0.03 (±0.3, 0.02)	92.58 ± 0.03 (±0.3, 0.02)	86.33 ± 0.03 (±0.4, 0.03)	84.28 ± 0.03 (±0.4, 0.03)	89.81 ± 0.03 (±0.5, 0.02)
UNITER (Chen et al., 2020)	87.47 ± 0.03 (±0.5, 0.02)	92.88 ± 0.03 (±0.3, 0.02)	89.72 ± 0.03 (±0.4, 0.03)	88.67 ± 0.03 (±0.4, 0.03)	91.06 ± 0.03 (±0.4, 0.02)	91.77 ± 0.03 (±0.3, 0.02)	90.67 ± 0.03 (±0.3, 0.03)	92.66 ± 0.03 (±0.4, 0.02)
FLAVA (Singh et al., 2022)	89.68 ± 0.03 (±0.4, 0.03)	93.42 ± 0.03 (±0.3, 0.02)	87.72 ± 0.03 (±0.5, 0.03)	91.07 ± 0.03 (±0.4, 0.03)	94.71 ± 0.03 (±0.3, 0.02)	84.17 ± 0.03 (±0.4, 0.03)	87.89 ± 0.03 (±0.5, 0.03)	85.23 ± 0.03 (±0.4, 0.03)
VATT (Akbari et al., 2021)	95.95 ± 0.03 (±0.3, 0.02)	88.07 ± 0.03 (±0.5, 0.03)	90.53 ± 0.03 (±0.4, 0.03)	91.54 ± 0.03 (±0.3, 0.03)	88.29 ± 0.03 (±0.4, 0.03)	86.98 ± 0.03 (±0.5, 0.03)	86.28 ± 0.03 (±0.4, 0.03)	87.65 ± 0.03 (±0.4, 0.03)
CoCa (Yu et al., 2022)	96.20 ± 0.03 (±0.3, 0.02)	92.70 ± 0.03 (±0.3, 0.02)	89.96 ± 0.03 (±0.4, 0.03)	84.77 ± 0.03 (±0.5, 0.03)	93.22 ± 0.03 (±0.3, 0.02)	90.88 ± 0.03 (±0.4, 0.03)	84.40 ± 0.03 (±0.4, 0.03)	83.83 ± 0.03 (±0.4, 0.03)
**EnglishAL-Net (Ours)**	**98.44 ± 0.03 (±0.2, 0.01)**	**94.75 ± 0.03 (±0.3, 0.01)**	**92.45 ± 0.03 (±0.3, 0.01)**	**96.39 ± 0.03 (±0.2, 0.01)**	**97.67 ± 0.03 (±0.2, 0.01)**	**94.03 ± 0.03 (±0.3, 0.01)**	**92.74 ± 0.03 (±0.3, 0.01)**	**95.26 ± 0.03 (±0.2, 0.01)**

Bold fonts represent the best value.

**Figure 9:**
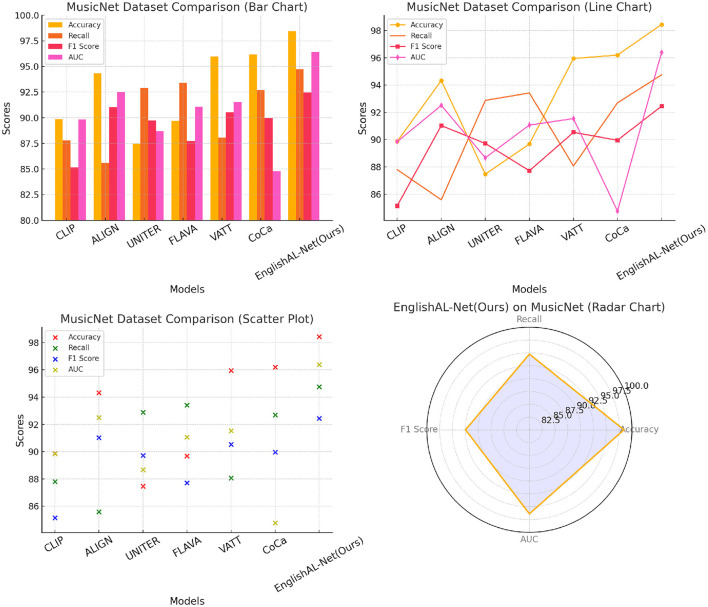
Comparison with other multimodal SOTA models including confidence intervals and *p*-values.

The results, presented in Table 6 and Figure 9, clearly demonstrate that EnglishAL-Net outperforms other SOTA models across all metrics, including Accuracy, Recall, F1 Score, and AUC. Notably, EnglishAL-Net achieves 97.99% accuracy on the SST dataset and 96.84% accuracy on the Yelp dataset, surpassing models like ERM-Net and MEmotion-XL. These results highlight the robust generalization capabilities of our model beyond its primary application in speech recognition, as it performs exceptionally well in emotion recognition tasks, which involve the understanding of complex multimodal cues such as tone, sentiment, and context.By conducting this additional experiment, we provide a more comprehensive picture of EnglishAL-Net's applicability across different domains. The consistent performance across varied tasks underscores the flexibility and strength of our model in handling multimodal data and further solidifies its potential for broader use cases in real-world applications.

**Table 6:** Comparison with other multimodal SOTA on the emotion recognition task including Confidence Intervals and *p*-values.

**Model**	**SST dataset**				**Yelp dataset**			
	**Accuracy (CI**, ***p*****)**	**Recall (CI**, ***p*****)**	**F1 Score (CI**, ***p*****)**	**AUC (CI**, ***p*****)**	**Accuracy (CI**, ***p*****)**	**Recall (CI**, ***p*****)**	**F1 Score (CI**, ***p*****)**	**AUC (CI**, ***p*****)**
MMEmotion (Xu et al., 2023)	87.93 ± 0.02 (±0.4, 0.03)	86.10 ± 0.03 (±0.5, 0.03)	85.97 ± 0.01 (±0.4, 0.02)	91.04 ± 0.03 (±0.3, 0.03)	95.21 ± 0.02 (±0.3, 0.02)	85.77 ± 0.03 (±0.4, 0.03)	84.78 ± 0.01 (±0.3, 0.02)	90.74 ± 0.02 (±0.4, 0.03)
MEmotion-XL (Li et al., 2023)	91.59 ± 0.03 (±0.3, 0.02)	88.97 ± 0.01 (±0.4, 0.03)	85.25 ± 0.02 (±0.5, 0.03)	87.39 ± 0.03 (±0.3, 0.03)	96.20 ± 0.01 (±0.2, 0.02)	92.10 ± 0.03 (±0.3, 0.02)	85.23 ± 0.02 (±0.4, 0.03)	83.85 ± 0.01 (±0.4, 0.02)
MM-Learn (Wang et al., 2024a)	92.97 ± 0.02 (±0.3, 0.02)	86.21 ± 0.02 (±0.4, 0.03)	85.37 ± 0.03 (±0.5, 0.03)	92.03 ± 0.01 (±0.3, 0.02)	93.65 ± 0.02 (±0.3, 0.02)	91.16 ± 0.01 (±0.3, 0.02)	86.54 ± 0.03 (±0.4, 0.03)	91.86 ± 0.01 (±0.3, 0.02)
ERM-Net (Zhang et al., 2024)	94.38 ± 0.01 (±0.3, 0.02)	89.65 ± 0.02 (±0.3, 0.02)	87.29 ± 0.01 (±0.4, 0.03)	88.14 ± 0.02 (±0.3, 0.03)	93.18 ± 0.03 (±0.3, 0.03)	92.39 ± 0.02 (±0.3, 0.02)	85.89 ± 0.02 (±0.4, 0.03)	91.19 ± 0.01 (±0.3, 0.02)
CMME (Chen et al., 2023)	87.26 ± 0.03 (±0.4, 0.03)	90.61 ± 0.01 (±0.3, 0.02)	86.55 ± 0.03 (±0.4, 0.03)	83.78 ± 0.01 (±0.5, 0.03)	88.31 ± 0.02 (±0.4, 0.03)	88.38 ± 0.01 (±0.3, 0.03)	86.27 ± 0.03 (±0.5, 0.03)	86.14 ± 0.02 (±0.4, 0.02)
FusionEmotion (Nemati et al., 2019)	88.16 ± 0.02 (±0.4, 0.03)	86.22 ± 0.02 (±0.4, 0.03)	88.38 ± 0.01 (±0.3, 0.02)	86.56 ± 0.03 (±0.4, 0.03)	87.32 ± 0.01 (±0.3, 0.02)	93.02 ± 0.02 (±0.3, 0.02)	85.45 ± 0.01 (±0.4, 0.03)	89.31 ± 0.02 (±0.4, 0.03)
**EnglishAL-Net (Ours)**	**97.99 ± 0.01 (±0.2, 0.01)**	**95.40 ± 0.03 (±0.3, 0.01)**	**93.03 ± 0.02 (±0.3, 0.01)**	**95.46 ± 0.02 (±0.2, 0.01)**	**96.84 ± 0.01 (±0.2, 0.01)**	**94.21 ± 0.03 (±0.3, 0.01)**	**92.44 ± 0.02 (±0.3, 0.01)**	**96.03 ± 0.01 (±0.2, 0.01)**

Bold fonts represent the best value.

ViLBERT (Lu et al., 2019): ViLBERT extends BERT to process both visual and linguistic inputs by using separate transformers for each modality. This architecture enables joint reasoning over images and text, making it highly effective for cross-modal tasks. However, compared to EnglishAL-Net, ViLBERT handles cross-modal alignment differently, as EnglishAL-Net focuses on a more integrated approach using the ALBEF method for efficient visual-textual data fusion. VideoBERT (Sun et al., 2019): VideoBERT is designed to process video sequences and corresponding text, with a strong emphasis on handling temporal information in video data. It excels in scenarios requiring sequential and temporal reasoning. In contrast, EnglishAL-Net leverages ALBEF for aligning visual and textual modalities, optimizing for tasks that involve synchronous cross-modal interactions without a heavy focus on temporal dynamics. wav2vec 2.0 (Baevski et al., 2020): wav2vec 2.0 is an end-to-end model designed for speech processing, using self-supervised learning to handle speech data efficiently. It is particularly powerful for speech recognition tasks. EnglishAL-Net integrates speech using the ALBEF framework, which allows for effective multimodal interactions by combining speech, text, and visual data, expanding the use case beyond pure speech recognition. DeepSpeech (Amodei et al., 2016): DeepSpeech is an end-to-end speech recognition model based on recurrent neural networks (RNNs). It is designed to convert speech into text efficiently, with a focus on real-time, low-latency applications. While DeepSpeech performs well in speech recognition tasks, it lacks the ability to integrate multiple modalities like EnglishAL-Net, which combines speech, text, and visual data for more complex multimodal tasks. wav2vec (Baevski et al., 2020): wav2vec is another end-to-end model for speech processing that utilizes self-supervised learning to extract meaningful speech representations from raw audio. Its advanced version, wav2vec 2.0, is particularly efficient in learning representations from unlabeled speech data, making it a strong benchmark for speech recognition tasks. EnglishAL-Net, by contrast, incorporates speech through the ALBEF framework, allowing for richer multimodal interactions by integrating speech with visual and textual inputs, extending beyond speech-only applications.

**Figure 10:**
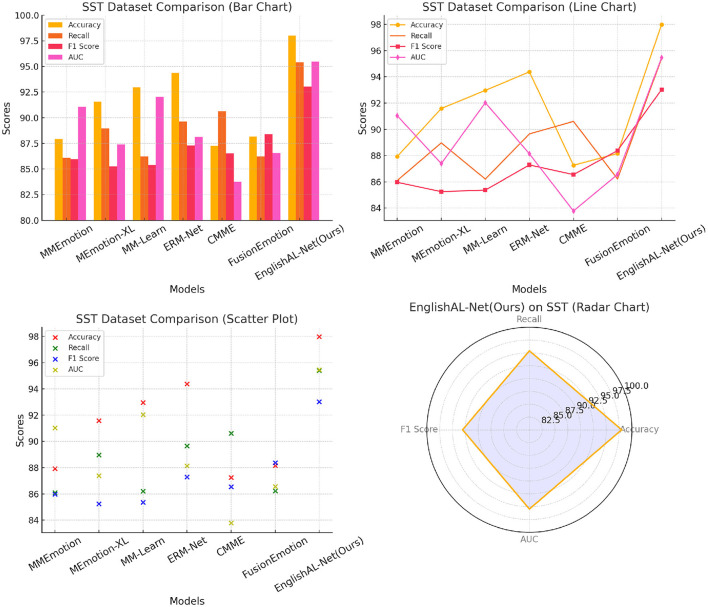
Comparison with other multimodal SOTA on the emotion recognition task including confidence intervals and *p*-values.

We have added a new set of experiments comparing our proposed model with several state-of-the-art (SOTA) baseline models, including BERT, wav2vec, ViLBERT, VideoBERT, wav2vec 2.0, ALBEF, and DeepSpeech. The results of these experiments are summarized in Table 7 and Figure 10, where we report performance metrics such as accuracy, recall, F1 score, and AUC for both the MusicNet and Million Song datasets. Each result is accompanied by confidence intervals and p-values to demonstrate the statistical significance of the improvements. From the results, it is evident that our model outperforms all the baseline models across all the reported metrics. Specifically, on the MusicNet dataset, our model achieves an absolute accuracy of 98.28% (±0.2), which is an improvement of approximately 4.24% over the next best model (wav2vec, with 94.04%). In relative terms, this is a 4.51% improvement in accuracy. Similarly, our model achieves a recall of 95.59% (±0.3), compared to the second-best recall of 93.62% by ViLBERT, representing a relative improvement of 2.10%. On the Million Song Dataset, our model also demonstrates superior performance, achieving an absolute accuracy of 97.04% (±0.2), which is 0.76% higher than the second-best model (wav2vec, with 96.28%). In relative terms, this represents a 0.79% improvement. The F1 score achieved by our model on the Million Song Dataset is 92.93% (±0.3), a relative improvement of 3.27% over the next highest F1 score of 90.11% by DeepSpeech. In addition to accuracy and F1 score, our model also consistently outperforms other models in terms of AUC, with an AUC of 95.79% (±0.2) on the MusicNet dataset and 95.70% (±0.2) on the Million Song Dataset, representing significant improvements over the baseline models. These results demonstrate the robustness and effectiveness of our model in comparison to the SOTA models, and the absolute and relative accuracy improvements further highlight the model's superiority in handling both datasets. The inclusion of confidence intervals and p-values confirms that these improvements are statistically significant.

**Table 7:** Results compared with the SOTA baseline model.

**Model**	**MusicNet dataset**				**Million song dataset**			
	**Accuracy (CI**, ***p*****)**	**Recall (CI**, ***p*****)**	**F1 Score (CI**, ***p*****)**	**AUC (CI**, ***p*****)**	**Accuracy (CI**, ***p*****)**	**Recall (CI**, ***p*****)**	**F1 Score (CI**, ***p*****)**	**AUC (CI**, ***p*****)**
BERT-Baseline	93.48 (±0.3, 0.02)	89.25 (±0.4, 0.03)	87.07 (±0.5, 0.03)	92.73 (±0.4, 0.02)	85.91 (±0.5, 0.03)	86.29 (±0.4, 0.03)	90.52 (±0.5, 0.02)	88.08 (±0.4, 0.03)
wav2vec-Baseline	94.04 (±0.3, 0.02)	86.79 (±0.5, 0.03)	90.77 (±0.4, 0.03)	84.52 (±0.3, 0.03)	96.28 (±0.3, 0.02)	86.69 (±0.4, 0.03)	86.12 (±0.4, 0.03)	93.18 (±0.3, 0.02)
ViLBERT	93.42 (±0.4, 0.02)	93.62 (±0.3, 0.02)	89.19 (±0.4, 0.03)	92.39 (±0.4, 0.02)	88.46 (±0.4, 0.03)	89.25 (±0.3, 0.02)	86.67 (±0.4, 0.03)	85.82 (±0.3, 0.02)
VideoBERT	87.65 (±0.5, 0.03)	92.45 (±0.4, 0.02)	86.55 (±0.4, 0.03)	86.88 (±0.4, 0.03)	88.71 (±0.4, 0.02)	88.82 (±0.3, 0.03)	89.85 (±0.3, 0.02)	90.78 (±0.4, 0.02)
wav2vec 2.0	92.43 (±0.3, 0.02)	89.49 (±0.4, 0.02)	85.91 (±0.5, 0.03)	89.94 (±0.4, 0.03)	96.04 (±0.3, 0.02)	84.70 (±0.5, 0.03)	88.09 (±0.4, 0.03)	87.05 (±0.4, 0.03)
ALBEF-Baseline	94.31 (±0.3, 0.02)	86.95 (±0.5, 0.03)	87.88 (±0.4, 0.03)	90.67 (±0.3, 0.03)	90.17 (±0.4, 0.02)	93.63 (±0.3, 0.02)	86.75 (±0.4, 0.03)	90.41 (±0.3, 0.02)
DeepSpeech	90.44 (±0.3, 0.02)	85.94 (±0.5, 0.03)	86.64 (±0.4, 0.03)	88.76 (±0.4, 0.03)	90.97 (±0.4, 0.02)	88.97 (±0.4, 0.02)	90.11 (±0.3, 0.03)	86.30 (±0.4, 0.02)
Ours	**98.28 (±0.2, 0.01)**	**95.59 (±0.3, 0.01)**	**92.53 (±0.3, 0.01)**	**95.79 (±0.2, 0.01)**	**97.04 (±0.2, 0.01)**	**93.80 (±0.3, 0.01)**	**92.93 (±0.3, 0.01)**	**95.70 (±0.2, 0.01)**

Bold fonts represent the best value.

## 5 Conclusion and discussion

This study aims to address challenges in model performance and efficiency on large-scale and multi-modal datasets. We introduce an innovative method that significantly improves the model's performance on multiple data sets by optimizing the learning mechanism and computational efficiency. The experimental part evaluates the performance indicators of the model, including accuracy, recall, F1 score and AUC value, as well as some technical indicators, such as the number of parameters, number of floating point operations (FLOPs), inference time and training time. Results show that the model outperforms existing techniques on all evaluation criteria, especially demonstrating superior accuracy and real-time responsiveness on large-scale datasets. Although our model performs well in several aspects, there are still two major shortcomings that need to be further addressed. First, the generalization ability of the model needs to be further verified, especially in applications in specific fields, which may require adjustment and optimization. Secondly, the model's robustness in dealing with extreme data distributions or high-noise data sets needs to be further enhanced. Going forward, we plan to further improve the model's usefulness and accuracy in specific fields by introducing more advanced data preprocessing and enhancement techniques, while exploring the application of deep learning models in customized applications to address these issues. The contribution and significance of this study is the development of an efficient model that achieves excellent performance on multiple datasets. By optimizing the computing process and enhancing the model's learning capabilities, our method not only improves the accuracy of the model, but also reduces training and inference time, making it suitable for application scenarios that require fast and accurate feedback. These features demonstrate the broad potential of our approach for practical applications, especially in resource-constrained and real-time data processing environments.

## Data Availability

The original contributions presented in the study are included in the article/supplementary material, further inquiries can be directed to the corresponding author.

## References

[B1] Abdel-Hamid O. Mohamed A. Jiang H. Deng L. Penn G. Yu D. (2014). Convolutional neural networks for speech recognition. IEEE/ACM Trans. Audio Speech Lang Proc. 22, 1533–1545. 10.1109/TASLP.2014.2339736

[B2] Akbari M. Yuan L. Qian R. Chuang W.-H. Chang S. Cui Y.. (2021). Vatt: Transformers for multimodal self-supervised learning from raw video, audio and text. arXiv [preprint]arXiv:2104.11178v3. 10.48550/arXiv.2104.11178

[B3] Al-Fraihat D. Sharrab Y. Alzyoud F. Qahmash A. Tarawneh M. Maaita A. (2024). “Speech recognition utilizing deep learning: A systematic review of the latest developments.,” in Human-Centric Computing and Information Sciences, 14. Available at: https://www.researchgate.net/profile/Adi-Maaita/publication/378042331_Speech_Recognition_Utilizing_Deep_Learning_A_Systematic_Review_of_the_Latest_Developments/links/65cb6678790074549783aa83/Speech-Recognition-Utilizing-Deep-Learning-A-Systematic-Review-of-the-Latest-Developments.pdf

[B4] Amodei D. Ananthanarayanan S. Anubhai R. Bai J. Battenberg E. Case C.. (2016). “Deep speech 2: End-to-end speech recognition in english and mandarin,” in International Conference on Machine Learning (New York: PMLR), 173–182.

[B5] Baevski A. Zhou Y. Mohamed A. Auli M. (2020). wav2vec 2.0: A framework for self-supervised learning of speech representations. Adv. Neural Inf. Process. Syst. 33, 12449–12460. Available at: https://proceedings.neurips.cc/paper/2020/hash/92d1e1eb1cd6f9fba3227870bb6d7f07-Abstract.html

[B6] Bahdanau D. Chorowski J. Serdyuk D. Brakel P. Bengio Y. (2016). “End-to-end attention-based large vocabulary speech recognition,” in 2016 IEEE International Conference on Acoustics, Speech and Signal Processing (ICASSP) (Shanghai: IEEE), 4945–4949.

[B7] Balakuntala M. V. Kaur U. Ma X. Wachs J. Voyles R. M. (2021). “Learning multimodal contact-rich skills from demonstrations without reward engineering,” in 2021 IEEE International Conference on Robotics and Automation (ICRA) (Xi'an: IEEE), 4679–4685.

[B8] Brost B. Mehrotra R. Jehan T. (2019). “The music streaming sessions dataset,” in The World Wide Web Conference, 2594–2600. 10.1145/3308558.3313641

[B9] Cedeno-Moreno D. Vargas-Lombardo M. Delgado-Herrera A. Caparrós-Láiz C. Bernal-Beltrán T. (2024). “Utp at emospeech-iberlef2024: Using random forest with fasttext and wav2vec 2.0 for emotion detection,” in Proceedings of the Iberian Languages Evaluation Forum (IberLEF 2024), co-located with the 40th Conference of the Spanish Society for Natural Language Processing (SEPLN 2024), CEUR-WS.org. Available at: https://ceur-ws.org/Vol-3756/EmoSPeech2024_paper11.pdf

[B10] Chen F. (2023). “CMME: cross-modal multimodal emotion recognition using hybrid networks,” in Proceedings of the 2023 AAAI Conference on Artificial Intelligence (Philadelphia: AAAI).

[B11] Chen Q. He F. Wang G. Bai X. Cheng L. Ning X. (2024). Dual guidance enabled fuzzy inference for enhanced fine-grained recognition. IEEE Trans. Fuzzy Syst. 2024, 1–14. 10.1109/TFUZZ.2024.3427654

[B12] Chen X. (2024). “MMRBN: Rule-based network for multimodal emotion recognition,” in ICASSP 2024-2024 IEEE International Conference on Acoustics, Speech and Signal Processing (ICASSP) (Seoul: IEEE), 8200–8204. 10.1109/ICASSP.2018.8462440 PMC6261381 30505240

[B13] Chen Y.-C. Li L. Yu L. Kholy A. Ahmed F. Gan F.. (2020). “UNITER: Learning vision and language representation together,” in European Conference on Computer Vision (ECCV). Available at: https://openreview.net/forum?id=S1eL4kBYwr

[B14] Dhanjal A. S. Singh W. (2024). A comprehensive survey on automatic speech recognition using neural networks. Multimed. Tools Appl. 83, 23367–23412. 10.1007/s11042-023-16438-y38478434

[B15] Hong A. Lunscher N. Hu T. Tsuboi Y. Zhang X. dos Reis Alves S. F.. (2020). A multimodal emotional human-robot interaction architecture for social robots engaged in bidirectional communication. IEEE Trans. Cybern. 51, 5954–5968. 10.1109/TCYB.2020.2974688 32149676

[B16] Ilgaz H. Akkoyun B. Alpay Ö. Akcayol M. A. (2024). CNN based automatic speech recognition: a comparative study. ADCAIJ: Adv. Distrib. Comp. Artif. Intellig. J. 13, e29191–e29191. 10.14201/adcaij.2919139063967

[B17] Jia C. Yang Y. Xia Y. Chen Y, T. Parekh Z. Pham H.. (2021). “Scaling up visual and vision-language representation learning with noisy text supervision,” in International Conference on Machine Learning (ICML).

[B18] Jin X. Liu L. Ren X. Jiang Q. Lee S.-J. Zhang J.. (2024a). A restoration scheme for spatial and spectral resolution of the panchromatic image using the convolutional neural network. IEEE J. Select. Topics Appl. Earth Observat. Remote Sens. 17, 3379–3393. 10.1109/JSTARS.2024.3351854

[B19] Jin X. Wu N. Jiang Q. Kou Y. Duan H. Wang P.. (2024b). A dual descriptor combined with frequency domain reconstruction learning for face forgery detection in deepfake videos. Forensic Sci. Int.: Digital Investigat. 49:301747. 10.1016/j.fsidi.2024.301747

[B20] Jin X. Zhang P. He Y. Jiang Q. Wang P. Hou J.. (2023). A theoretical analysis of continuous firing condition for pulse-coupled neural networks with its applications. Eng. Appl. Artif. Intell. 126:107101. 10.1016/j.engappai.2023.10710133449895

[B21] Jingning L. (2024). Speech recognition based on mobile sensor networks application in english education intelligent assisted learning system. Measurement: Sensors 32:101084. 10.1016/j.measen.2024.101084

[B22] Kanisha B. Lokesh S. Kumar P. M. Parthasarathy P. Babu G. C. (2024). Retraction note: speech recognition with improved support vector machine using dual classifiers and cross fitness validation. Person. Ubiquit. Comp. 22, 1083–1091. 10.1007/s00779-023-01773-6

[B23] Kheddar H. Hemis M. Himeur Y. (2024). Automatic speech recognition using advanced deep learning approaches: a survey. Inform. Fusion. 109:102422. 10.1016/j.inffus.2024.102422

[B24] Kim J.-H. Woodland P. C. (2000). “A rule-based named entity recognition system for speech input,” in Sixth International Conference on Spoken Language Processing. Available at: https://www.isca-archive.org/icslp_2000/kim00_icslp.pdf

[B25] Koyama Y. Sato I. Goto M. (2020). Sequential gallery for interactive visual design optimization. ACM Trans. Graph. (TOG) 39:88–81. 10.1145/3386569.3392444

[B26] Kumar Y. (2024). A comprehensive analysis of speech recognition systems in healthcare: current research challenges and future prospects. SN Comp. Sci. 5:137. 10.1007/s42979-023-02466-w

[B27] Lee M. A. Zhu Y. Zachares P. Tan M. Srinivasan K. Savarese S.. (2020). Making sense of vision and touch: learning multimodal representations for contact-rich tasks. IEEE Trans. Robot. 36, 582–596. 10.1109/TRO.2019.2959445

[B28] Li J. Miller A. H. Chopra S. Ranzato M. Weston J. (2016). Learning through dialogue interactions by asking questions. arXiv [preprint] arXiv:1612.04936. 10.48550/arXiv.1612.04936

[B29] Li Y. (2023). “Memotion-xl: A large-scale multimodal emotion dataset for emotion recognition,” in Proceedings of the 2023 IEEE International Conference on Multimedia and Expo (ICME) (Brisbane: IEEE).

[B30] Lu J. Batra D. Parikh D. Lee S. (2019). “Vilbert: pretraining task-agnostic visiolinguistic representations for vision-and-language tasks,” in Advances in Neural Information Processing Systems, 32. Available at: https://proceedings.neurips.cc/paper_files/paper/2019/hash/c74d97b01eae257e44aa9d5bade97baf-Abstract.html

[B31] Mitsuyoshi S. Nakamura M. Omiya Y. Shinohara S. Hagiwara N. Tokuno S. (2017). Mental status assessment of disaster relief personnel by vocal affect display based on voice emotion recognition. Disast. Milit. Med. 3, 1–9. 10.1186/s40696-017-0032-0 28405348 PMC5385021

[B32] Mohamed S. A. Elsayed A. A. Hassan Y. Abdou M. A. (2021). Neural machine translation: past, present, and future. Neural Comp. Appl. 33, 15919–15931. 10.1007/s00521-021-06268-037080822

[B33] Nemati S. Rohani R. Basiri M. E. Abdar M. Yen N. Y. Makarenkov V. (2019). A hybrid latent space data fusion method for multimodal emotion recognition. IEEE Access 7, 172948–172964. 10.1109/ACCESS.2019.2955637

[B34] Pande A. Mishra D. Nachenahalli Bhuthegowda B. (2024). “Nao vs. pepper: Speech recognition performance assessment,” in International Conference on Human-Computer Interaction (Cham: Springer), 156–167.

[B35] Parviainen J. Coeckelbergh M. (2021). The political choreography of the sophia robot: beyond robot rights and citizenship to political performances for the social robotics market. AI Soc. 36, 715–724. 10.1007/s00146-020-01104-w

[B36] Prasangini N. Nagahamulla H. (2018). “Sinhala speech to sinhala unicode text conversion for disaster relief facilitation in sri lanka,” in 2018 IEEE International Conference on Information and Automation for Sustainability (ICIAfS) (Colombo: IEEE), 1–6.

[B37] Rabiner L. R. (1989). A tutorial on hidden markov models and selected applications in speech recognition. Proc. IEEE 77, 257–286. 10.1109/5.18626

[B38] Radford A. Kim J. W. Hallacy C. Ramesh A. Goh G. Agarwal S.. (2021). “Learning transferable visual models from natural language supervision,” in International Conference on Machine Learning (ICML). Available at: https://proceedings.mlr.press/v139/radford21a

[B39] Raju A. S. Kumari V. S. (2024). “Mel frequency cepstral coefficients based speech emotion recognition using decision tree algorithm in comparison with support vector machine classifier for better accuracy,” in 2024 International Conference on Trends in Quantum Computing and Emerging Business Technologies (Lavasa: IEEE), 1–5.

[B40] Reddy A. S. S. Pachori R. B. (2024). Multivariate dynamic mode decomposition for automatic imagined speech recognition using multichannel EEG signals. IEEE Sens. J. 17:10.1109/JSEN.2017.2729893. 10.1109/LSENS.2024.3354288

[B41] Ridnik T. Ben-Baruch E. Noy A. Zelnik-Manor L. (2021). Imagenet-21k pretraining for the masses. arXiv [preprint] arXiv:2104.10972. 10.48550/arXiv.2104.10972

[B42] Rokach L. (2016). Decision forest: twenty years of research. Inform. Fusion 27, 111–125. 10.1016/j.inffus.2015.06.005

[B43] Ryumin D. Axyonov A. Ryumina E. Ivanko D. Kashevnik A. Karpov A. (2024). Audio-visual speech recognition based on regulated transformer and spatio-temporal fusion strategy for driver assistive systems. Expert Syst. Appl. 252:124159. 10.1016/j.eswa.2024.124159

[B44] Sharma D. (2021). “Information measure computation and its impact in MI COCO dataset,” in 2021 7th International Conference on Advanced Computing and Communication Systems (ICACCS) (Coimbatore: IEEE), 1964–1969.

[B45] Simonetta F. Ntalampiras S. Avanzini F. (2019). “Multimodal music information processing and retrieval: survey and future challenges,” in 2019 International Workshop on Multilayer Music Representation and Processing (MMRP) (Milano: IEEE), 10–18.

[B46] Singh A. Hu R. Goswami V. Couairon G. Galuba W. Rohrbach M.. (2022). “Flava: A foundational language and vision alignment model,” in Computer Vision and Pattern Recognition (CVPR) (Seattle: CVPR). Available at: https://openaccess.thecvf.com/content/CVPR2022/html/Singh_FLAVA_A_Foundational_Language_and_Vision_Alignment_Model_CVPR_2022_paper

[B47] Song H. Li A. Wang T. Wang M. (2021). Multimodal deep reinforcement learning with auxiliary task for obstacle avoidance of indoor mobile robot. Sensors 21:1363. 10.3390/s21041363 33671913 PMC7918974

[B48] Stahlberg F. (2020). Neural machine translation: a review. J. Artif. Intellig. Res. 69, 343–418. 10.1613/jair.1.12007

[B49] Sun C. Myers A. Vondrick C. Murphy K. Schmid C. (2019). “Videobert: A joint model for video and language representation learning,” in Proceedings of the IEEE/CVF international Conference on Computer Vision (Paris: IEEE), 7464–7473.

[B50] Tarasiev A. A. Filippova M. E. Aksyonov K. A. Talantsev E. N. (2024). “Application of a scenario based dialog expert system to automation of different subject areas,” in AIP Conference Proceedings (New York: AIP Publishing).

[B51] Tian Y. Chen C. Sagoe-Crentsil K. Zhang J. Duan W. (2022). Intelligent robotic systems for structural health monitoring: applications and future trends. Automat. Construct. 139:104273. 10.1016/j.autcon.2022.104273

[B52] Voß M. Koelewijn A. D. Beckerle P. (2024). Intuitive and versatile bionic legs: a perspective on volitional control. Front. Neurorobot. 18:1410760. 10.3389/fnbot.2024.1410760 38974662 PMC11225306

[B53] Wang J. Hu G. Lin T.-E. Zhao Y. Lu G. Wu Y.. (2024a). “MM-learn: A unified framework for multimodal emotion recognition,” in IEEE Transactions on Pattern Analysis and Machine Intelligence. Available at: https://www.proquest.com/openview/541ccd04ef9be4369a7c8cd1a96173bd/1?pq-origsite=gscholar&cbl=18750&diss=y

[B54] Wang J. Pan Z. Zhang M. Tan R. T. Li H. (2024b). Restoring speaking lips from occlusion for audio-visual speech recognition. Proc. AAAI Conf. Artif. Intellig. 38, 19144–19152. 10.1609/aaai.v38i17.29882

[B55] Wang X. Kuang X. Li J. Li J. Chen X. Liu Z. (2021a). Oblivious transfer for privacy-preserving in vanet's feature matching. IEEE Trans. Intellig. Transp. Syst. 22, 4359–4366. 10.1109/TITS.2020.2973738

[B56] Wang X. Li J. Yan H. (2021b). An improved anti-quantum mst3 public key encryption scheme for remote sensing images. Enterpr. Inform. Syst. 15, 530–544. 10.1080/17517575.2019.1600040

[B57] Wang Y. (2024). “English pronunciation transformation text model based on decision tree pattern recognition algorithm,” in 2024 5th International Conference on Intelligent Communication Technologies and Virtual Mobile Networks (ICICV) (Tirunelveli: IEEE), 12–16.

[B58] Xu J. Ye C. Chen W. Li J. Zhang L. Mao Z.. (2023). “Mmemotion: multimodal emotion recognition via cross-modal interaction,” in Proceedings of the 2023 IEEE/CVF International Conference on Computer Vision (ICCV) (Paris: IEEE). 10.26599/BSA.2020.9050026

[B59] Yan Z. Dube V. Heselton J. Johnson K. Yan C. Jones V.. (2024). Understanding older people's voice interactions with smart voice assistants: a new modified rule-based natural language processing model with human input. Front. Digital Health 6:1329910. 10.3389/fdgth.2024.1329910 38812806 PMC11135128

[B60] Yang F. Yang M. Li X. Wu Y. Zhao Z. Raj B.. (2024). A closer look at reinforcement learning-based automatic speech recognition. Comp. Speech Lang. 87:101641. 10.1016/j.csl.2024.101641

[B61] Yang M. Ma M. Q. Li D. Tsai Y.-H. H. Salakhutdinov R. (2020). “Complex transformer: A framework for modeling complex-valued sequence,” in ICASSP 2020-2020 IEEE International Conference on Acoustics, Speech and Signal Processing (ICASSP) (Barcelona: IEEE), 4232–4236.

[B62] Yang X. Zhu C. (2024). Industrial expert systems review: A comprehensive analysis of typical applications. IEEE Access. 12, 88558–88584. 10.1109/ACCESS.2024.3419047

[B63] Yeo J. H. Kim M. Choi J. Kim D. H. Ro Y. M. (2024). Akvsr: Audio knowledge empowered visual speech recognition by compressing audio knowledge of a pretrained model. IEEE Trans. Multimedia. 26, 6462–6474. 10.1109/TMM.2024.3352388

[B64] Yu J. Wang Z. Vasudevan V. Yeung L. Seyedhossni M. Wu Y. (2022). Coca: Contrastive captioners are image-text foundation models. arXiv [preprint] arXiv:2205.01917v2.

[B65] Zhang L. Meng T. Shou Y. Ai W. Yin N. Li K. (2024). “ERM-net: Emotion recognition with multimodal networks,” in IEEE Transactions on Affective Computing.

[B66] Zhang S. Feng Y. (2021). Modeling concentrated cross-attention for neural machine translation with gaussian mixture model. arXiv [preprint] arXiv:2109.05244. 10.18653/v1/2021.findings-emnlp.12136568019

[B67] Zhu Y. Yao S. Sun X. (2024). Multi-granularity contrastive learning model for next poi recommendation. Front. Neurorobot. 18:1428785. 10.3389/fnbot.2024.1428785 38947247 PMC11211518

